# Targeted inhibition of BET proteins in HPV16-positive head and neck squamous cell carcinoma reveals heterogeneous transcriptional responses

**DOI:** 10.3389/fonc.2024.1440836

**Published:** 2024-09-05

**Authors:** Aakarsha Rao, Milan S. Stosic, Chitrasen Mohanty, Dhruthi Suresh, Albert R. Wang, Denis L. Lee, Kwangok P. Nickel, Darshan S. Chandrashekar, Randall J. Kimple, Paul F. Lambert, Christina Kendziorski, Trine B. Rounge, Gopal Iyer

**Affiliations:** ^1^ Department of Human Oncology, University of Wisconsin School of Medicine and Public Health, University of Wisconsin, Madison, WI, United States; ^2^ Department of Life Sciences and Health, Faculty of Health Sciences, OsloMet - Oslo Metropolitan University, Oslo, Norway; ^3^ Department of Microbiology and Infection Control, Akershus University Hospital, Lørenskog, Norway; ^4^ Department of Biostatistics and Medical Informatics, University of Wisconsin-Madison, Madison, WI, United States; ^5^ Department of Biomedical Engineering, University of Wisconsin-Madison, Madison, WI, United States; ^6^ McArdle Laboratory for Cancer Research, Department of Oncology, University of Wisconsin School of Medicine and Public Health, Madison, WI, United States; ^7^ University of Wisconsin Carbone Cancer Center, Madison, WI, United States; ^8^ Molecular and Cellular Pathology, Department of Pathology, University of Alabama at Birmingham, Birmingham, AL, United States; ^9^ Centre for Bioinformatics, Department of Pharmacy, University of Oslo, Oslo, Norway; ^10^ Norwegian Institute of Public Health, Cancer Registry of Norway, Oslo, Norway

**Keywords:** BRD4, HPV16, HNSCC, RNA-seq, E6, E7

## Abstract

Human papillomaviruses (HPV), most commonly HPV16, are associated with a subset of head and neck squamous cell carcinoma (HNSCC) tumors, primarily oropharyngeal carcinomas, with integration of viral genomes into host chromosomes associated with worse survival outcomes. We analyzed TCGA data and found that HPV+ HNSCC expressed higher transcript levels of the bromodomain and extra terminal domain (BET) family of transcriptional coregulators. The role of BET protein-mediated transcription of viral-cellular genes in the viral-HNSCC genomes needs to be better understood. Using a combination of TAME-Seq, qRT-PCR, and immunoblot analyses, we show that BET inhibition downregulates E6 and E7 significantly, with heterogeneity in the downregulation of viral transcription across different HPV+ HNSCC cell lines. Chemical BET inhibition was phenocopied with the knockdown of BRD4, mirroring the downregulation of viral E6 and E7 expression. We found that BET inhibition directly downregulated c-Myc and E2F expression and induced CDKN1A (p21) expression, leading to a G1-cell cycle arrest with apoptotic activity. Overall, our studies demonstrate that BET inhibition regulates both E6 and E7 viral and key cellular cell cycle regulator E2F gene expression and cellular gene expression in HPV-associated HNSCC and highlight the potential of BET inhibitors as a therapeutic strategy for this disease while also underscoring the importance of considering the heterogeneity in cellular responses to BET inhibition.

## Introduction

Papillomaviruses are a diverse group of non-enveloped, double-stranded DNA viruses known for their specific affinity for squamous epithelial tissues across various host species (1). Their life cycle is intricately intertwined with the differentiation program of host epithelial cells, commencing with an initial infection of basal epithelial cells and culminating in a productive phase as these cells differentiate and migrate toward the surface, ultimately releasing new virions without causing cell lysis (2). Human papillomaviruses (HPV) possess a compact genome encoding nine proteins, categorized as early (E) and late (L) proteins (3). The early proteins E1, E2, E1^E4, E5, E6, E7 and E8^E2 play crucial roles in the viral life cycle and pathogenesis (4). E1 and E2 are essential for viral DNA replication, with E1 serving as a DNA helicase (5) and E2 regulating transcription while acting as a segregation factor during cell division (6). E1^E4 plays a role in virus release and can disrupt the host cell’s intermediate filament network (7). E5 enhances the proliferation of infected cells and contributes to immune evasion by downregulating MHC class I molecules (8). The oncoproteins E6 and E7 have profound multifunctional roles in pathogenesis, the most well-known being the ability of E6 to target p53 for degradation (9), and E7 to inactivate the retinoblastoma protein (Rb), leading to cell cycle dysregulation (10). Given that E6 and E7 expression is critical for proliferation and its significance in reactivation of dormant tumor suppressive pathways (11–13), its downregulation can provide an alternative route for reducing anti-proliferative pathways in HPV-associated cancers (14).

HPV genomes can exist in both extrachromosomal and integrated forms. While the extrachromosomal form is essential for the productive viral life cycle, integration into the host genome is often associated with cellular transformation, making this process pivotal in HPV-induced carcinogenesis (15). In the initial stages of infection, HPV exists as a circular, extrachromosomal DNA (16). This form of the viral genome is maintained in host cells through the function of E1 and E2. During carcinogenesis, HPV DNA can integrate into the host genome (17). Integration often leads to increased expression of E6 and E7 oncogenes, propelling the cell toward malignancy (15). However, E6 and E7 are insufficient to cause cancer; additional genomic alterations and co-factors are typically required (18).

Given the molecular features, HPV is a significant etiological factor in head and neck squamous cell carcinomas (HNSCC), particularly in oropharyngeal cancers (OPSCC). HPV plays a significant role in oropharyngeal squamous cell carcinoma (OPSCC), with prevalence rates varying from 22.4% to 45.8% of cases. HPV16 is the predominant type in HPV(+) OPSCC. The incidence of HPV-positive OPSCC shows marked geographic variation, with North America and Europe exhibiting higher rates than Asia and South America. For instance, in the United States, about 60% of OPSCC cases are HPV16(+), while Europe sees 31%, and Brazil only 4%. This regional disparity underscores the importance of considering geographical factors in understanding the epidemiology and management of HPV-related OPSCC (19–22).

Therapeutic interventions targeting E6 and E7 proteins, exogenous to human cells and consistently expressed in HPV-positive tumors, represent ideal therapeutic targets (23). A recent and promising area of therapeutic intervention is the class of bromodomain and extra terminal (BET) inhibitors, which target proteins containing bromodomains, including BRD4 (24). BRD4, a member of the BET family, plays pivotal roles in normal physiological processes, particularly in regulating gene transcription. By recognizing acetylated histones (25), BRD4 facilitates the recruitment of positive transcription elongation factor b (P-TEFb) to chromatin (26), thus promoting the elongation phase of transcription and influencing cell cycle progression (27), DNA damage response (28, 29), and cellular differentiation (30).

BRD4 plays a crucial role in the HPV viral life cycle (23, 30–35). Previous studies have reported BRD4 to be overexpressed and this correlates with poor prognosis in various solid cancers (36–39). BRD4’s interaction with episomal HPV genomes is well-documented. It interacts with the viral E2 protein, essential for maintaining the extrachromosomal state of the HPV genome. This interaction is crucial for the segregation of HPV genomes during cell division and regulation of viral transcription (31). In the case of integrated HPV genomes, BRD4 continues to play a role in the transcriptional regulation of HPV genes due to its broader roles in chromatin organization and gene transcription through its association with enhancers and super-enhancers (32). However, viral integration leading to super-enhancer formation is not universal and is influenced by specific cellular programs and cell types (33), underscoring the importance of the contextual environment of HPV’s integration. While BRD4 regulation in cervical cancer lines has been described extensively (34), very little is known in the context of integrated HPV HNSCC tumors. In patients with HPV16-associated HNSCC (HPV16 is the HPV genotype most commonly found in HNSCC), the presence of integrated HPV-16 is associated with poorer survival outcomes (35) compared to those cancers with extrachromosomal viral genomes (36).

This study investigated the impact of the pan-BET inhibitor JQ1 on viral transcription in seven HPV16-positive HNSCC cell lines harboring various genetic alterations (37). Our findings demonstrate JQ1 can inhibit expression of E6 and E7 oncogenes as well as the cellulat oncogene c-MYC across the cell lines, suggesting BET inhibitors may be effective in treating HPV+ HNSCC. The study also reveals dominant G1 cell-cycle arrest across most cell lines and the complex role of E2F gene family members and its target genes, highlighting the role of E2F as an alternative target as mechanism for cell-cycle arrest.

## Materials and methods

### Cell culture

UD: SCC2, UM: SCC47, UPCI: SCC90, UM: SCC104, 93VU147T, UPCI: SCC152, and UPCI: SCC154 cell lines were obtained from the NCI-Head and Neck SPORE biobank (University of Wisconsin-Madison). All cell lines were cultured in Dulbecco’s Modified Eagle Medium (DMEM) containing 4.5g/L glucose, L-glutamine, and sodium pyruvate, supplemented with 10% fetal bovine serum (FBS). Cells were maintained at 37°C in a humidified incubator with 5% (vol/vol) CO2 and 95% (vol/vol) air. All cell lines were tested and found negative for mycoplasma and were authenticated by short tandem repeat (STR) profiling performed by LabCorp.

### Southern blot

Assessment of the integration status of the HPV-positive HNSCC cell lines was characterized for each cell line and digested with 7 µg of total genomic DNA at 37°C overnight (20 hours) with either *EcoRV* (New England BioLabs) or *Bam HI.* HPV-positive HNSCC samples and λ HindIII marker and standards were loaded on a 1X TAE gel apparatus for gel electrophoresis at 30V overnight, followed by EtBr stain and destaining. The gel underwent denaturation and neutralization washes and was transferred overnight (20 hours) to a Hybond membrane (Amersham). The membrane was subjected to UV crosslink with a Strata Linker on auto crosslink. The ^32^P radiolabeled HPV16 genotype-specific single-stranded oligonucleotides probe was incubated in a Techne hybridization tube in a Techne hybridizer HB-1D incubator at 48°C. Membrane was subsequently washed with Church hybridization buffer followed by exposure on a Molecular Dynamics cassette overnight (24 hours). The radio-labelled blot was imaged with a Typhoon 8610 imager (Molecular Dynamics).

### Cell viability assays

HNSCC cell lines were treated with dimethyl sulfoxide (DMSO) as a vehicle control, 0.5 μM (+)-JQ1 (APExBIO), or 0.5 μM (-)-JQ1 (APExBIO) for 96 hours. Live cells in each condition were counted using a Bio-Rad TC20 digital cell counter with trypan blue exclusion. Cell numbers were normalized to the vehicle-treated group for all experiments. Cells were treated with 500 nM (+)-JQ1 or (-)-JQ1. 24 and 48 hours post-treatment, 100 µl/well of room temperature Caspase-Glo 3/7 reagent (Promega) was added to the treated and control cells. Luminescence was measured using a CLARIOstar microplate reader (BMG Labtech, Software version: 5.21 R2, Firmware version: 1.15).

### JQ1 treatments and cell viability determination

To determine the half-maximal inhibitory concentrations (IC50s) for each HNSCC cell line, cells were plated into 96-well plates at 1500-4500 cells/well and treated with various concentrations of (+)-JQ1 (range: 10 nM to 10 μM) for 96 hours. Cell viability was measured by adding 10 µL of PrestoBlue Cell Viability Reagent (Thermo Fisher) to each well containing 100 µL of medium and incubating at 37°C for 1 and 4 hours. Plates were read using a CLARIOstar microplate reader (BMG Labtech, Software version: 5.21 R2, Firmware version: 1.15) with excitation/emission at 535/590 nm. Raw absorbance values were background-corrected by subtracting the average absorbance value of medium-only wells. Cell survival rates at each (+)-JQ1 concentration were calculated by normalizing to the DMSO-treated control signal. Data were curve-fitted using the sigmoidal dose-response equation (Y = Bottom + (Top-Bottom)/(1 + 10^((LogIC_50_-X)*HillSlope))) in OriginLab software (OriginLab) to determine IC_50_ values.

### Lentiviral transduction

MISSION lentiviral-based shRNA vector collections (Sigma Aldrich, St. Louis, MO, USA) were used for long-term silencing of BRD4. Briefly, 1.6×104 cells were cultured in 96-well plates and incubated for 18-20 hours. After media removal, hexadimethrine bromide (8 μg/ml) was added, and effective multiplicity of infection (MOI) of lentiviral particles was established. Knockdown clones were identified using [TRCN0000199427], [TRCN0000318771], and [TRCN0000196576] (V-591). Following an 18-20 hour incubation at 37˚C, puromycin (1.0 µg/ml) was added, and resistant clones were selected and grown.

### Quantitative RT-PCR

All cell lines were pre-treated with 0.5 µM (+)-JQ1 or (-)-JQ1 (vehicle control) for 30 minutes and 24 hours before RNA extraction. Samples were collected using TRIzol reagent at 30 minutes and 24 hours post-treatment. RNA was isolated using chloroform extraction, ethanol wash, phase-lock separation, and DNase I treatments. First-strand cDNA was synthesized using a High-Capacity RNA-to-cDNA Kit (Applied Biosystems). Quantitative PCR reactions were set up using PowerUp SYBR Green Master Mix (Applied Biosystems) and performed on a Bio-Rad CFX96 machine. The cycling profile was set to 95^0^C – 5 min; 95^0^C – 15 sec; 60^0^C – 30 sec – 40 cycles followed by melt curve analyses from 65 - 95^0^C at an increment of 0.5^0^C. Primers are listed in **Table 1**. Ct values were determined using CFX Maestro 1.1 software (Bio-Rad), and the delta-delta Ct method was used to calculate the relative mRNA expression of each target gene relative to the housekeeping gene (58).

**Table 1:** Quantitative RT-PCR primers.

Primer	Forward	Reverse
**TBP**	TATAATCCCAAGCGGTTTGC	GCTGGAAAACCCAACTTCTG
**UBC**	CCTTATCTTGGATCTTTGCCTTG	GATTTGGGTCGCAGTTCTTG
**BRD4**	GGAAGAGGACAAGTGCAAGC	GCTTCAGGGTCTCAAAGTCG
**cMYC**	TCCTCGGATTCTCTGCTCTC	TCTTCCTCATCTTCTTGTTCCTC
**E2F1**	ATGTTTTCCTGTGCCCTGAG	ATCTGTGGTGAGGGATGAGG
**HPV16E2**	ACAGACCTACGTGACCATATAGA	CCCATTTCTCTGGCCTTGTAATA
**HPV16E6**	ATGTTTCAGGACCCACAGG	CCCGAAAAGCAAAGTCATATACC
**HPV16E7**	CGGACAGAGCCCATTACAAT	TCTTCCAAAGTACGAATGTCTACG

### Flow cytometry

All cell lines were cultured in high-glucose DMEM supplemented with 10% FBS in a humidified incubator at 37°C with 5% CO2. Cells were plated at a density of 0.5x106 cells per 10 cm dish and treated with 0.5 µM (+)-JQ1 or DMSO (vehicle control) for 30 min, 24, and 48 hours to assess the effect on cell cycle progression. Following incubation, cells were harvested using Trypsin-EDTA (Corning), rinsed with 1X PBS, and fixed in 70% ethanol. Cells were then stained with propidium iodide (PI) (0.5 mg/ml, BioLegend) in a staining buffer containing PBS, DNA-free RNase (10 mg/ml), 1% Triton-X (Sigma), and autoclaved water. Cell cycle progression was analyzed using a Thermo Fisher Attune Flow Cytometer (4-laser, 14-color cytometer) with FSC=90V, SSC=280V, and PI=290V (yellow YL2 channel). Data were analyzed, and histograms were generated using ModFit software (ModFit LT V5.0.9), while bar graphs were prepared using OriginLab software.

### Cell lysate preparation and Western blot analysis

Whole cell lysates were prepared by solubilizing cell pellets (10 million cells per treatment) in RIPA buffer (G-Bioscience) supplemented with 1X Protease and Phosphatase Inhibitor Cocktail (Thermo Scientific78442) and 1X Phosphatase Inhibitor Cocktails 1 and 2 (Apex Bio K1012+K1013). Cells were sonicated at 10% amplitude for 10 seconds using a Fisher Scientific Sonic Dismembrator (Model 500). After a 30-minute incubation on a rotator at 4°C, cell samples were centrifuged at 14,000 rpm for 15 minutes at 4°C. Samples were diluted using 4X Laemmli Sample Buffer (Bio-Rad) containing 2-mercaptoethanol and DI-water to achieve a uniform concentration of 2 µg/µl after protein quantitation using the BCA assay. Proteins (30 µg per well) were separated on NuPAGE 4-12% Bis-Tris 26-well Midi gels (Invitrogen) using 1X MES running buffer. Novex Sharp Pre-Stained Protein Standard and SeeBlue Plus2 Pre-Stained Protein Standard (Life Technologies) were used as molecular weight markers. Proteins were transferred onto 0.2 µm nitrocellulose membranes (Thermo Scientific) using the Bio-Rad Trans-Blot Turbo Transfer System. Membranes were stained with freshly prepared Ponceau stain to confirm protein transfer and blocked with 3% bovine serum albumin (BSA) - Fraction V, heat-shock treated (Fisher Scientific) containing 0.5% sodium azide to prevent contamination. Membranes were probed with the following primary antibodies dilutions: CDKN2A (CST 2947), total p53 (CST 2527), total Rb (CST 9313), P-Rb (S807-811) (CST 9308), P-Rb (S795) (CST 9301) – all CST antibodies were used at 1:1000 dilution, BRD4 (Abcam 128874) *– 1:2000* E2F1 (Santa Cruz sc-251), c-Myc (Santa Cruz sc-40) – *1:2000* P-Rb (S780) (Sigma-Aldrich R6275) – *1:1000*, GAPDH (Proteintech HRP60004*) – 1:20000*, HPV16 E6 (GeneTex GTX132686), and HPV16 E7 (GeneTex GTX133411) *– 1:500*. Mouse IgG (CST 7076S) and rabbit IgG (CST 7074S) were used as secondary antibodies. SuperSignal West Femto Maximum Sensitivity Substrate (Thermo Scientific) was used for detection. Blots were imaged using the Li-Cor FC machine, and images were analyzed and quantified using Image Studio software (version 4.0.21). Each sample was normalized to its respective housekeeping loading control from the same day.

### RNA-sequencing

Total RNA (1 µg) was used for library preparation using the NEBNext Ultra II Directional RNA Library Prep Kit (New England Biolabs, Ipswich, MA, USA). Poly-A selection and cDNA synthesis were performed according to the NEB protocol, with adaptors diluted at a 1:30 ratio instead of the recommended 1:10 ratio. Size selection was performed using SPRI Select Beads (Beckman Coulter, Indianapolis, IN, USA) with in-house calibration values. The cDNA was amplified with 22 cycles of PCR, and 2x150 paired-end sequencing was performed at a depth of 30 million reads at Novogene (Davis, Sacramento).

### RNA-seq data analysis

Read mapping visualization on the HPV16 genome was conducted following the TaME-seq2 protocol (38). Initially, raw reads were trimmed with cutadapt (v3.4) and remapped to the human (GRCh38.p14) and HPV16 (PaVE) reference genomes via STAR (v2.7.9a) and HISAT2 (v2.2.1). Sorted BAM files, containing only HPV16 mapping information from HISAT2 mapping, were then processed with bcftools mpileup (v1.12) to compile mapping statistics for each position of the HPV16 reference. Read counts at each position underwent normalization to reads per million mapped reads (RPM). The normalized read counts were visualized using ggplot2 (v3.5.0) in RStudio (v2023.12.1). The gene-by-sample count matrix was calculated via RSEM-1.3.0 (39) using information from STAR mapping. Genes with an average expression less than 1 were filtered out. Median-by-ratio normalization (40) was conducted to obtain a normalized expression matrix. Differential expression (DE) analysis was performed between JQ1+ and JQ1- samples using DESeq2-1.40.2 (41, 59). DESeq2 adjusts the p-values using the Benjamini and Hochberg method to account for multiple testing. Genes with an adjusted p-value less than or equal to 0.01 and an absolute value of log2 fold change greater than or equal to 1 were selected as significantly differentially expressed genes.

TCGA Statistical analysis: Gene expression values for BRD genes (TPM) from HNSCC TCGA data were compared between HPV-positive and HPV-negative groups. Statistical significance was determined using unpaired t-tests. A p-value < 0.05 was considered statistically significant. All analyses was done using R package.

## Results

### JQ1 exhibits anti-proliferative effects and induces apoptosis in HPV16-positive HNSCC cell lines

**Figure 1:**
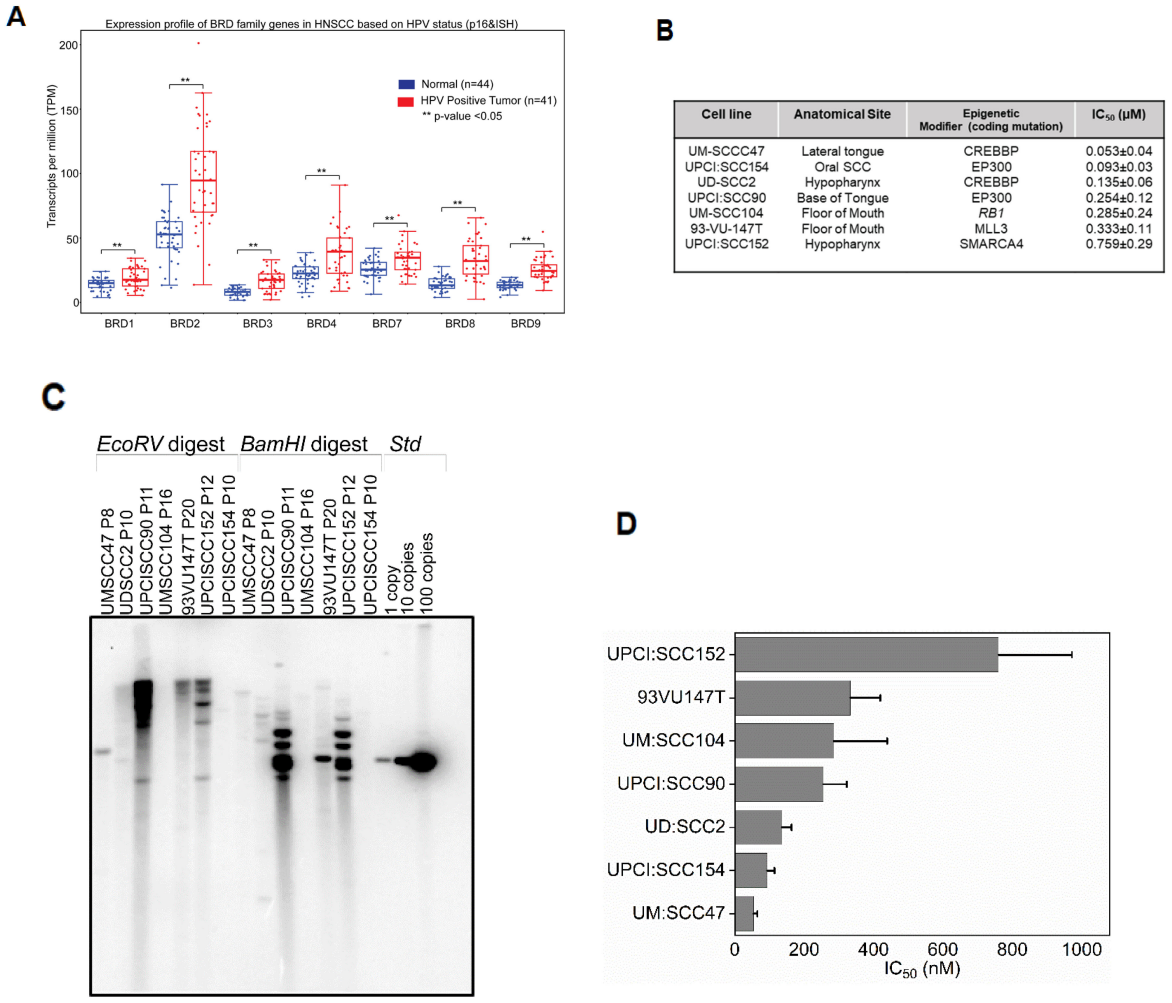
pan-BET inhibitor JQ1 treatment inhibits the growth of integrated HPV-associated head and neck squamous carcinoma cells (HNSCC) **(A)** TCGA data derived from UALCAN denotes BRD mRNA expression containing both bromodomains BD1 and BD2 associated with HPV status profiled through p16 and viral *in situ* hybridization from 41 patients was significantly higher compared to 44 normal patients. Y-axis represents transcripts per million (TPM), and Student’s t-test ** P<0.05. **(B)** IC_50_ values for seven HPV integrated HNSCC cell lines. Viable cells were determined using presto blue after treating cells with increasing concentration of JQ1 for 72 h. Error bars denote the SDs of independent experiments using six wells per dose. **(C)** Southern blot hybridization of restriction digested of seven HPV cell lines. Control HPV ~ 8 kb plasmid with copy numbers 1, 10, and 100 was loaded to validate the presence of integrated HPV DNA **(D)** IC_50_ values for seven HPV-integrated HNSCC cell lines. Viable cells were determined using presto blue after treating cells with increasing concentration of JQ1 for 72 h. Error bars denote the SDs of independent experiments using six wells per dose.

To examine the expression profile of *BRD4* in HPV16-positive HNSCC, we evaluated the expression of BET genes using the publicly available, curated TCGA dataset, UALCAN (42), which included 44 normal (adjacent tissue to the tumor) and 41 HPV-positive HNSCC patients. As shown in **Figure 1A**, BRD1, BRD2, BRD3, BRD4, and BRD7-9 were significantly upregulated in tumor samples expressing high levels of p16, a surrogate biomarker for HPV-positive cancers (43), and the presence of HPV DNA through *in situ* hybridization (44), compared to normal samples (p<0.05). Given these differences in expression, we hypothesized that HPV-associated tumors might exhibit a transcriptional reliance on BET proteins by enhancing transcription of viral and host oncogenic drivers, including HPV oncogenes E6 and E7 and/or c-MYC, a BRD4-dependent host oncogene (45), in HPV-positive HNSCC. Considering the differential expression of BRD4 between tumor and normal tissues, we further propose that t BET inhibition might serve as a potential alternative to conventional genotoxic agents like cisplatin, which induces non-specific DNA cross-links (46, 47). To evaluate this hypothesis, we employed seven established HPV-associated HNSCC cell lines differing in tumor origin and mutations in the coding regions of genes encoding epigenetic modifiers that regulate transcription (**Figure 1B**) (37). These cell lines are reported to harbor integrated forms of HPV16 (48), which we verified by Southern analysis using probes for HPV16 DNA (**Figure 1C** **).**

**Figure 2:**
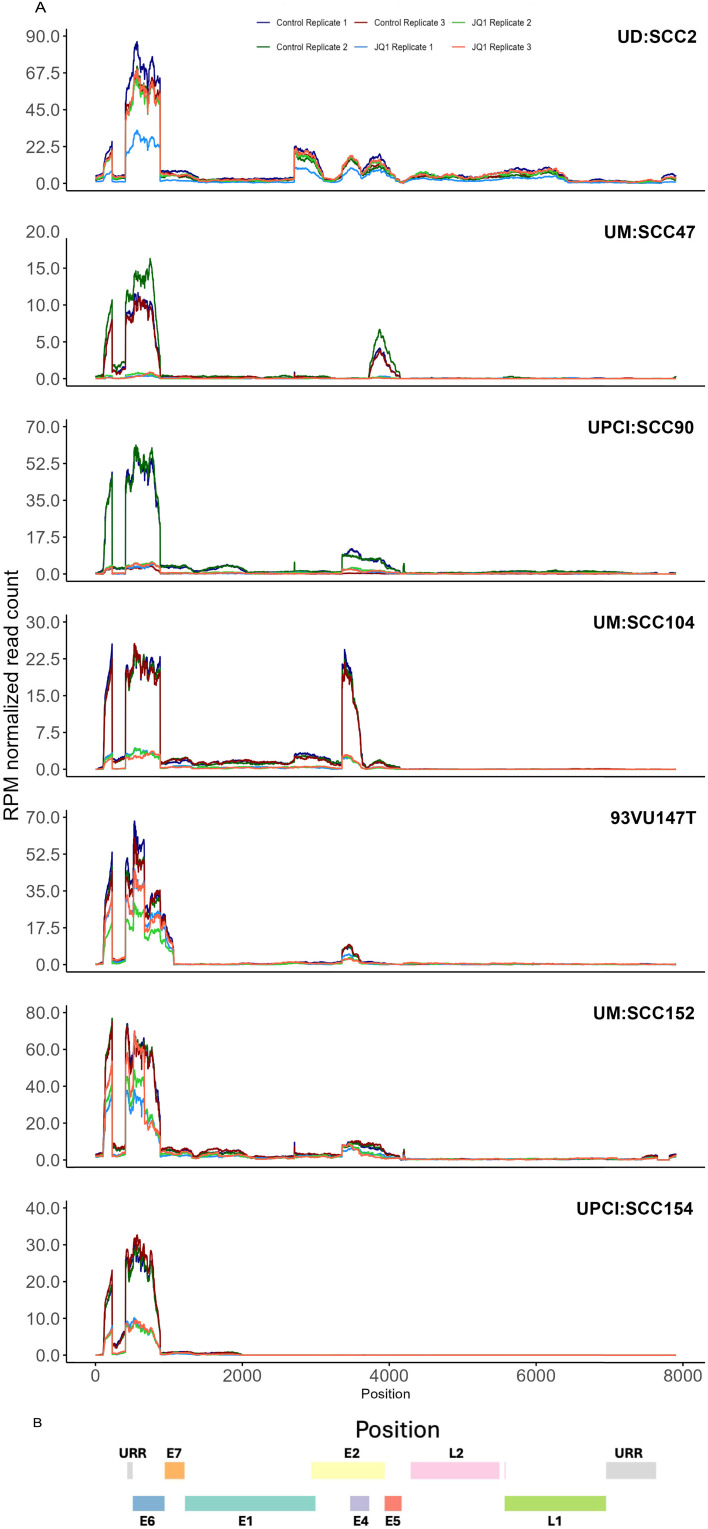
TAME-Seq analyses of viral RNA transcripts from HNSCC cell lines. **(A)** HPV coverage plots of expression of viral RNA genes at nucleotide resolution in untreated (control) and treated with JQ1. Each panel represents a different cell line indicated at the top. The x-axis represents the genomic position along the HPV16 genome, and the y-axis represents the reads per million normalized read counts. The coverage profiles are depicted as line plots, with different colors representing replicate samples within the same treatment condition. **(B)** The color-coded bar illustrates the genomic organization of the HPV16 genome, with different colors representing the different viral genes or regions URR (grey), E7 (orange), E6 (blue), E1 (teal), E2 (yellow), E4 (light purple), E5 (maroon), L2 (pink), L1 (green).

**Figure 3:**
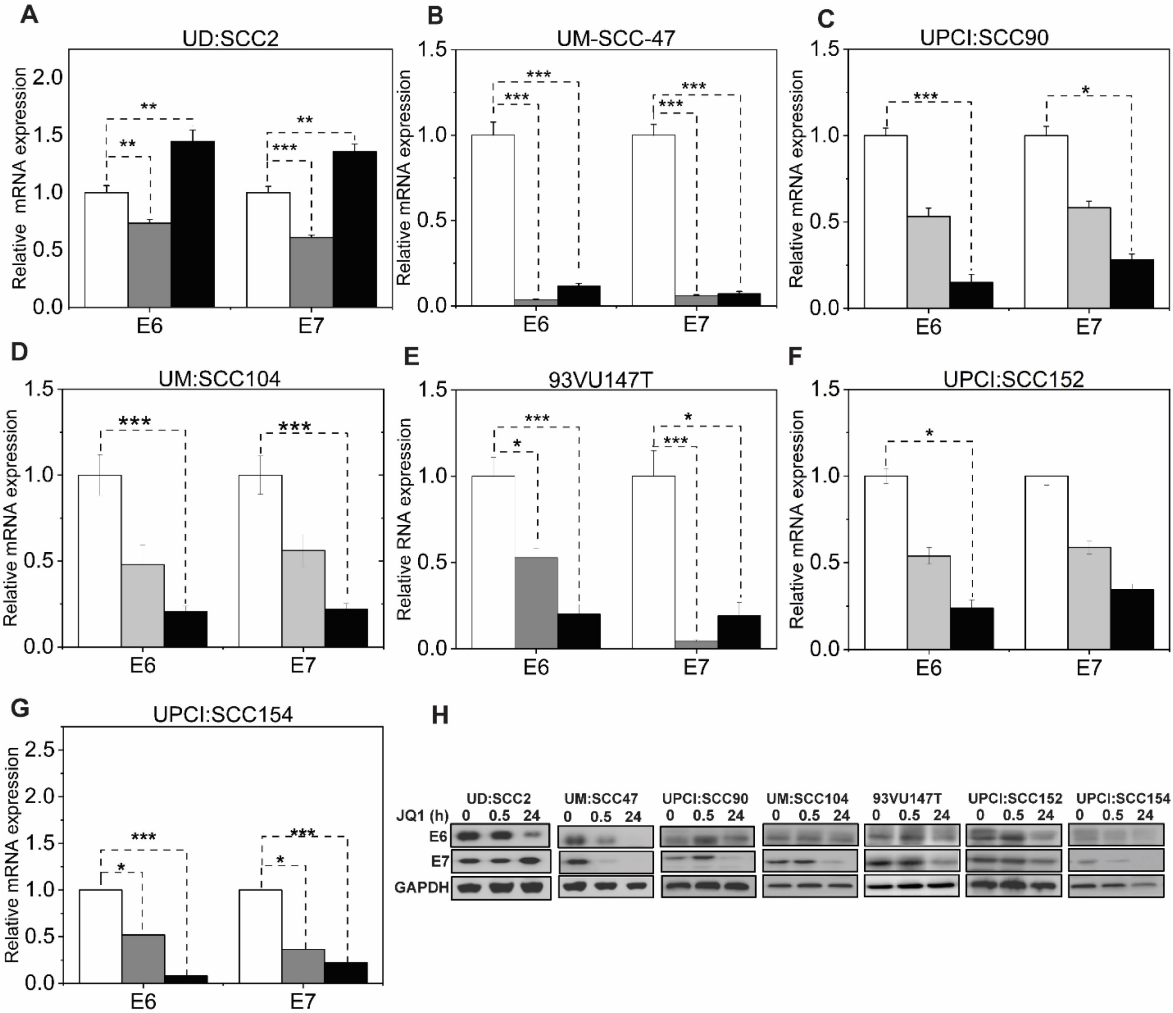
pan-BET inhibitor activity downregulates viral RNA and protein levels in selective HPV-associated HNSCC cells. **(A–G)** Validation of RNA-seq viral gene expression was quantified through transcript levels of viral E6, and E7 qRT-PCR in JQ1-treated cell lines at 500 nM concentration and normalized to vehicle JQ1 enantiomer at 30 minutes and 24 hours. RNA extraction was performed from three biological experiments, and gene expression was analyzed with three technical triplicates per experiment. Data are presented as the average ratio of viral E6, and E7 RNA levels for each cell line relative to vehicle control (mean ± SEM). Asterisks denote the level of statistical significance - (*P < 0.05, **P < 0.01, ***P < 0.005; two-tailed t-test). **(H)** Viral E6 and E7 protein levels in JQ1-treated HPV-positive HNSCC cell lines. Cells treated with JQ1 and its enantiomer (vehicle) at 30 min and 24 h lysates were immunoblotted with anti-E6 and E7 antibodies; GAPDH serves as a loading control.

We determined the efficacy of pan-BET inhibitor, JQ1(+), on the growth of these HPV16-positive HNSCC cell lines. JQ1(+) is known to selectively bind to the conserved bromodomains BD1 and BD2 of BRD4 with nanomolar affinities, thereby blocking its interaction with histones and consequently modulating transcription through protein-protein interactions (24). To assess the anti-proliferative potential of JQ1(+)- in HPV-positive cell lines, we used the control inactive enantiomer, JQ1-(-). These cell lines exhibited IC_50_ values ranging from 53 to 786 nM after 72 hours of treatment with JQ1(+) (**Figure 1D** **),** and the residual fits confirmed the accuracy of the IC50 values (**Supplementary Table S1** **).** Experiments showed that the inactive enantiomer JQ1(-) failed to affect the proliferation of UM: SCC47 and UD: SCC2 cell lines at 500 nM, for which JQ1(+) had IC_50_ of 53 and 135 nM (**Figure 1B**), respectively, as determined by the trypan blue assay (**Supplementary Figure S1A**). To better understand the mode of JQ1-induced growth inhibition, we undertook time-course experiments focusing on apoptosis by measuring Caspase3/7 activity. All tested cell lines demonstrated a marked increase in apoptosis relative to vehicle controls (**Supplementary Figure S1B**). JQ1 treatment promotes E6 and E7 down-regulation. Comprehensive HPV16 transcriptomics coverage analysis across all samples using the TAME-Seq bioinformatics pipeline (38) revealed expression of viral RNAs at nucleotide resolution, enabling reliable evaluation of viral gene expression, integration patterns, and the effects of JQ1 treatment (**Supplementary Tables S2**, **S3**). As shown in **Figure 2**, all seven cell lines displayed preferential expression of the early HPV16 oncogenes, E6 and E7, which are driven by the early promoter (P_97_ in HPV16), compared to other viral early genes, consistent with integration disrupting expression of most other viral genes. With JQ1 treatments (yellow shades), the reduced levels in the expression of the E6 and E7 oncogenes compared to the control replicates (brown, purple, and blue lines) indicated that E6 and E7 expression was reproducibly decreased across replicates in some but not all cell lines (**Figure 2**) with UM: SCC47, UPCI: SCC90, UM: SCC104 and UPCI: SCC154 displaying the strongest reductions in levels of E6 and E7 transcripts, with 93VU147T and UPCI-SCC152 showing more modest decreases. UD: SCC2 did not show a decrease in 2 of the 3 replicates. To confirm these data, we performed targeted qRTPCR of the E6 and E7 mRNAs (**Figures 3A–G**). Likewise, Western blot analyses for E6 and E7 proteins further confirmed these RNA analyses with UM: SCC47, UPCI: SCC90, UM: SCC104, and UPCI: SCC154 displaying the strongest reductions in levels of E6 and E7 proteins (**Figure 3H**). These qRT-PCR and immunoblot analyses also confirmed that only UD: SCC2 failed to show reduced levels of E6 and E7 containing transcripts or E6 and E7 proteins in response to JQ1(+) treatment. It is important to note that the detection of HPV16 E6 and E7 proteins presents significant challenges due to the limitations of currently available antibodies. These antibodies are known to exhibit variability in specificity and sensitivity, often resulting in non-specific binding and lot-to-lot variations. In our study, we have optimized the use of E6 and E7 antibodies through extensive validation and careful interpretation of results. To validate the JQ1 effects on downregulating E6 and E7 gene expression, we utilized shRNA-mediated stable knockdown of BRD4 in UM: SCC47 cells. The subsequent decrease in E6, and E7 expression was confirmed with BRD4 knockdown (**Supplementary Figure S2**). This finding not only corroborates the phenocopying effects observed with JQ1 chemical inhibition but also reinforces the central role of BRD4 as a transcriptional co-regulator of these viral genes.

Given the known roles of E6 and E7 in promoting cell cycle progression through their interactions with key cell cycle regulators (49), we sought to investigate the downstream effects of JQ1-mediated E6 and E7 downregulation on c-Myc and E2F family members, which are critical factors in cell cycle control.

### JQ1 downregulates cMyc with cell cycle dynamics

**Figure 4:**
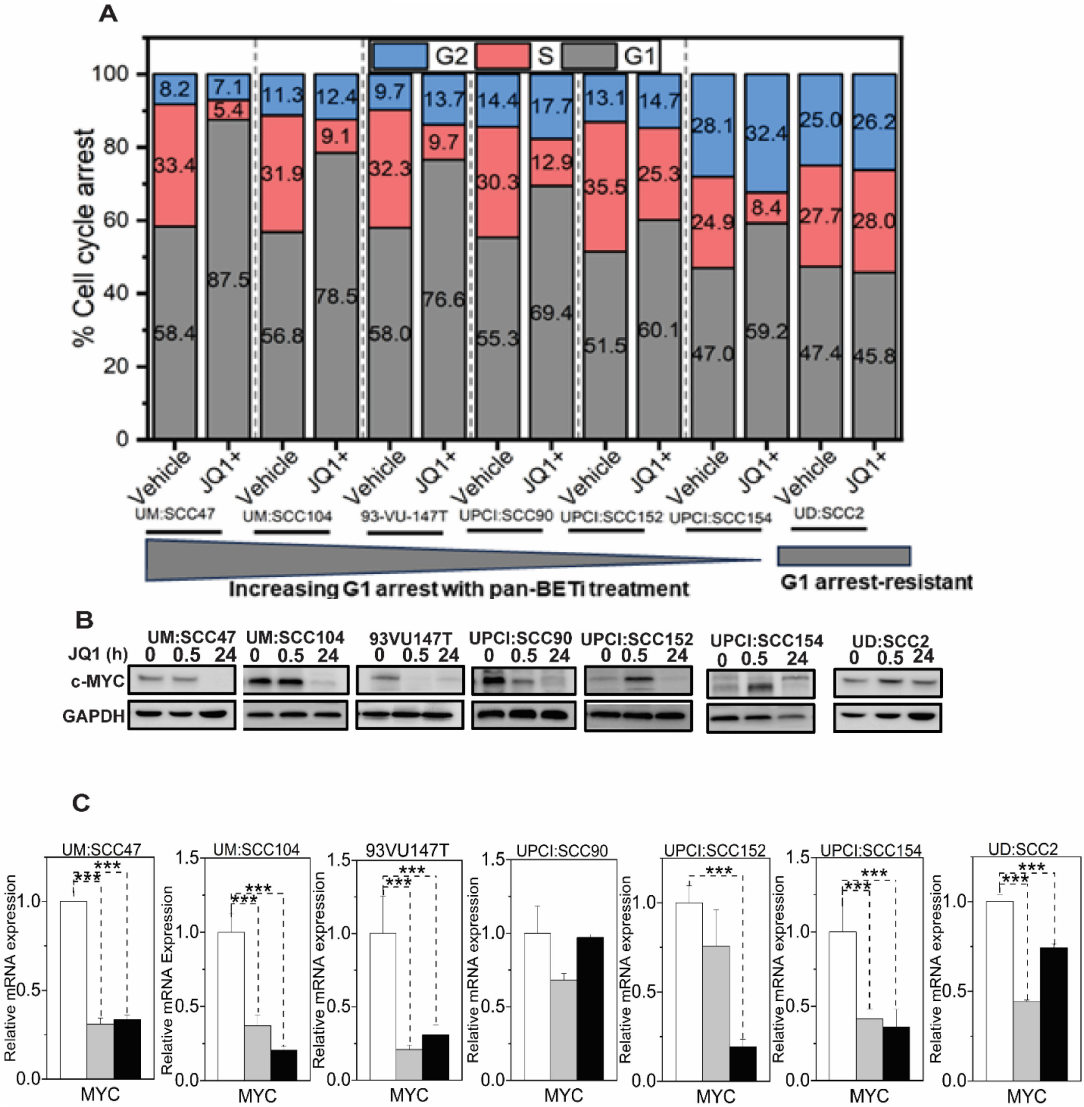
JQ1 treatment induces G1 cell cycle arrest and downregulates c-Myc in HPV-associated HNSCC cell lines **(A)** Cell-cycle analysis of seven integrated HPV-positive HNSCC cells treated with 500 nM for 24h and analyzed by propidium iodide flow cytometry. Stacked bar graphs show the percentage of cells in different cell cycle phases. Cell percentages were determined using OriginLab software, representing the mean values from three independent experiments. **(B)** c-Myc protein levels in seven integrated HPV-positive HNSCC cell lines treated with 500 nM JQ1. Cells were treated with JQ1 and its inactive enantiomer for 30 min and 24 hours, lysed, and probed with anti-cMyc antibody. GAPDH serves as a loading control. **(C)** qRT-PCR evaluated c-MYC RNA levels in cells treated for 30 min and 24 hours with 500 nM JQ1 and its vehicle control enantiomer JQ1. Mean fold change ±SEM (n=9) from three biological experiments. Asterisks denote the level of statistical significance - ( ***P < 0.005; two-tailed t-test).

Our findings reveal that JQ1 treatment in HPV-positive HNSCC leads to significant c-Myc downregulation, which correlates with G1 cell cycle arrest in most cell lines tested. This suggests that c-Myc modulation may contribute to the cellular response to BET inhibition in this context, despite c-Myc not being traditionally considered a primary oncogenic driver in HPV-associated HNSCC. Flow cytometry analysis of propidium iodide-stained cells at 24 hours post-treatment revealed a notable G1 phase cell cycle arrest in six out of seven cell lines: UM: SCC47 (87.5%), UM: SCC104 (78.5%), 93VU147T (76.6%), UPCI: SCC90 (69.4%), UPCI: SCC152 (60%), and UPCI: SCC154 (59.2%), compared to the corresponding inactive enantiomer treatments. Strikingly, UD: SCC2 showed no G1 phase restriction, with cell cycle distribution in G1 (45.8%), S (28%), and G2 (26.2%) phases (**Figure 4A**). Consistent with the proposed mechanism of JQ1 modulating the cell cycle through the c-Myc axis, we observed significant downregulation of c-MYC mRNA levels, ranging from 0.15 to 0.75-fold compared to controls, in UM: SCC47 and UD: SCC2 cells at 30 minutes and 24 hours post-JQ1 treatment (**Figure 4B**), followed by c-Myc downregulation at the protein level (**Figure 4C**). In addition to c-Myc, we also investigated the effects of JQ1 treatment on other key cell cycle regulators, such as p53 and p21, in the context of E6 downregulation in two of the contrasting cell-cycle phenotypes, UM: SCC47, G1-arrest sensitive and UD: SCC2, G1- arrest resistant and HPV-negative (Tu-138, D562, and FaDu) HNSCC cell lines. The E6-mediated degradation of p53 is a well-characterized mechanism in HPV-related cancers (39). As such, the observed increase in p53 protein levels upon JQ1-mediated downregulation of E6 protein at 24 h in UM: SCC47 and UD: SCC2 cells is consistent with the expected disruption of this interaction. However, this increase in p53 protein (**Supplementary Figure S3**) was not observed in HPV-negative HNSCC cell lines (Tu-138, D562, and FaDu), suggesting that the effect is specific to JQ1-mediated E6 downregulation (**Supplementary Figure S3**). Interestingly, p21 levels were upregulated by JQ1 treatment at 24 h in both HPV-positive – (UM: SCC47 and UD: SCC2) cells and HPV-negative – (Tu-138, D562, and FaDu) HNSCC cell lines (**Supplementary Figure S3**), suggesting that JQ1 can modulate p21 levels through both p53-dependent and independent mechanisms.

**Figure 5:**
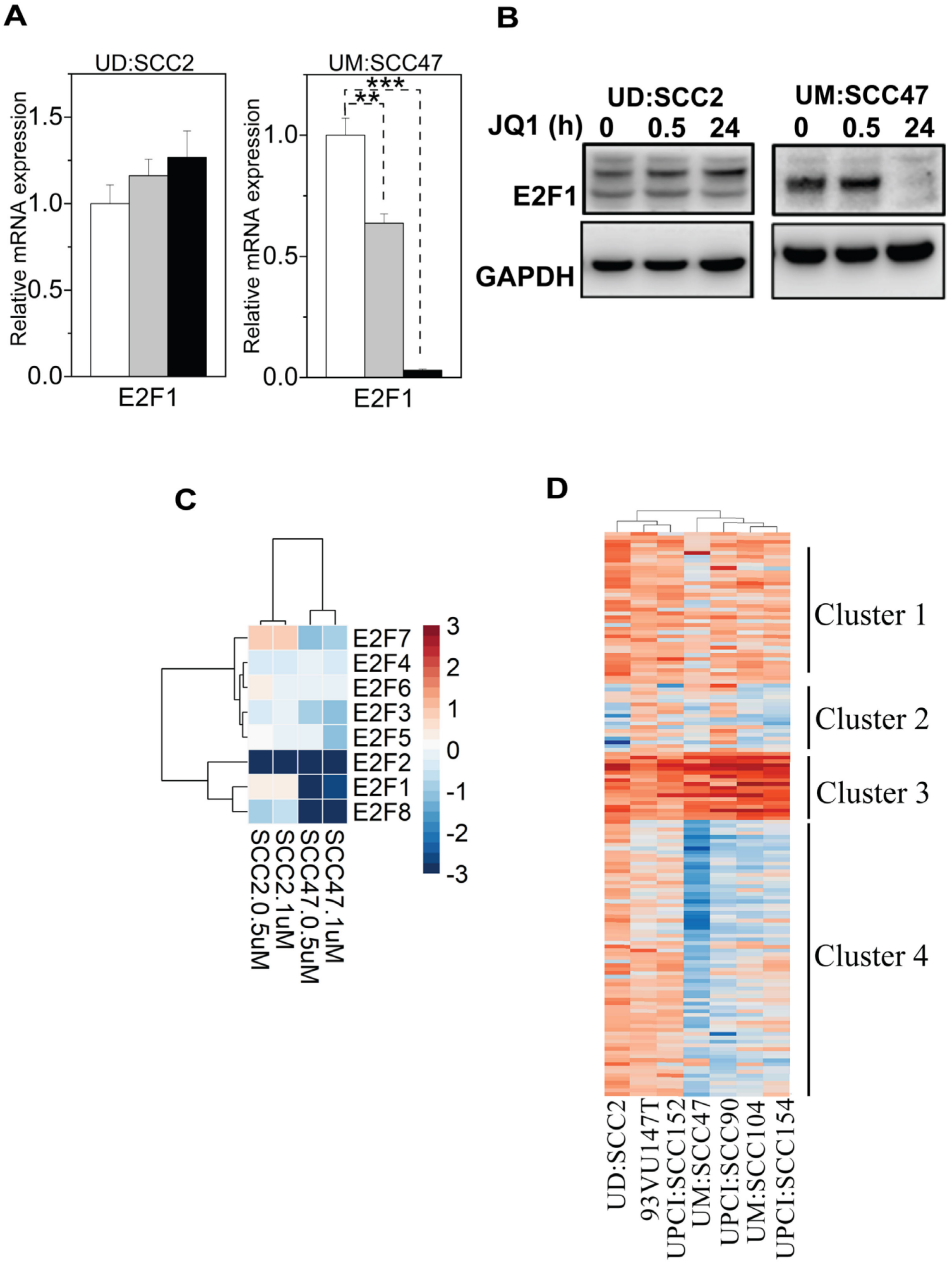
Expression and regulation of E2F1 in UD-SCC2 and UM-SCC47 cell lines upon treatment with JQ1. **(A)** qPCR analysis of E2F1 gene expression in UD: SCC2 (G1 arrest-resistant) and UM:SCC47 (G1 arrest-sensitive) cells following JQ1 treatment at 30 minutes and 24 hours. Data represent the mean ± SEM from triplicate biological experiments (n=9), showing the relative expression of E2F1 compared to the enantiomer control for each cell line. **P < 0.01, ***p < 0.001 compared to control. **(B)** Western blot analysis of E2F1 protein levels in UD: SCC2 and UM: SCC47 cell lines treated with JQ1 at 0, 0.5, and 24 hours. GAPDH serves as a loading control. **(C)** Heatmap showing differential expression of E2F family members (E2F1-E2F8) in a dose-response manner to JQ1 treatment across four HPV+ HNSCC cell lines. The color scale represents a log2 fold change in expression relative to control, with blue indicating downregulation and red indicating upregulation. Data shown for treatments with 500 nM (0.5 µM) and 1 µM JQ1 for 24 hours. **(D)** Hierarchical k-means clustering of differentially expressed genes of E2F targets obtained through DESeq2 analyses across all cell lines treated with 500 nM JQ1 in triplicate. The significant threshold was set to a log fold change of 2 and p-values ≤ 0.05.

Given the role of E2F transcription factors as downstream effectors of the pRb pathway, which is often disrupted by E7 in HPV-related cancers (46), we next investigated the dependency of E2F-transcriptional programs on BET protein regulation. Again, we focused on the contrasting cell lines UD: SCC2 and UM: SCC47. which showed different cell-cycle arrest responses. First, we identified that JQ1 treatment in UM: SCC47 cells led to a more pronounced reduction of E2F1 RNA and protein levels (nearly 95%) compared to UD: SCC2 (**Figures 5A, B**), suggesting a role for BET proteins to regulate E2F1 expression. Next, RNA-seq analysis of E2F members (E2F1-E2F8) at two JQ1 concentrations (500 nM and 1 µM) for 24 hours revealed a marked downregulation of E2F1, 2, and 8 in UM: SCC47, with E2F2 showing the most significant decrease in UD: SCC2 and an increase in E2F7(**Figure 5C**). The dose-dependent effects of JQ1 on E2F family members may contribute to the contrasting cell-cycling states in these two cell lines. Finally, we analyzed the E2F target genes (**Figure 5D**, **Supplementary Table S4**). Hierarchical k-means clustering across seven HNSCC cell lines revealed diverse expression profiles of E2F target genes grouped into four clusters ((**Figure 5D**). Clusters 1 and 2 encompass genes associated with cell cycle progression, DNA replication, and regulators such as CDKN1A (p21) and MDM2. Clusters 3 and 4 were distinguished by the increased expression of DNA damage response (DDR) genes and cell-cycle regulators, particularly in UD: SCC2, 93VU147T, and UPCI: SCC152 cell lines. This stratification implies a complex interplay of cell-cycle and DDR pathways influenced by JQ1 treatment, highlighting the variability in cellular responses and the potential for targeted therapies based on the specific molecular profiles of HPV-positive HNSCCs.

## Discussion

Our study characterizes the transcriptional changes elicited by JQ1, a BET inhibitor within HPV-positive HNSCC cell lines. JQ1 is a pan-BET inhibitor that competitively binds to the bromodomains of BET family proteins, including Brd2, Brd3, and Brd4, disrupting their interaction with acetylated histones and inhibiting transcriptional activation (24, 50). The pan-BET inhibitory activity of JQ1 is due to the high sequence and structural similarity of the bromodomains across these proteins (51). Investigating the individual roles of Brd2, Brd3, and Brd4 in cellular processes and disease pathogenesis is crucial for understanding the therapeutic potential and limitations of JQ1 and other BET inhibitors (45, 52, 53), as well as guiding the development of more selective inhibitors targeting specific BET family members (28). The rationale for utilizing BET inhibitors as a potential treatment option for HPV-positive tumors is supported by the analysis of The Cancer Genome Atlas (TCGA) data, which revealed elevated expression and activity of Brd2, Brd3, and Brd4 in HPV-positive HNSCC tumors compared to normal tissue.

Furthermore, JQ1 treatments resulted in the downregulation of E6 and E7 expression levels in six of the seven cell lines, an effect mirrored by BRD4 knockdown, indicating that BRD4 plays a pivotal role in the transcriptional regulation of these viral genes. This was consistent with the regulatory patterns seen in cervical cell line models (54), supporting the broad applicability of these findings. A key finding was the G1-cell cycle arrest induced by JQ1 in most cell lines, correlated with the downregulation of E6 and c-Myc and the upregulation of p21, indicating a reconfigured E6-p53-p21 axis. Studies have demonstrated that JQ1 treatment induces p21, contributing to its anti-leukemic activity in acute myeloid leukemia (AML) cells (55), and causes growth arrest and apoptosis in triple-negative breast cancer, where p21 plays a role in cell cycle arrest (56). A key finding of our study is the consistent downregulation of the E2F gene family and its downstream targets across multiple HPV-positive HNSCC cell lines in response to JQ1 treatment. This common transcriptional response emerges as a critical mechanism underlying the anti-tumor effects of BET inhibition in this cancer type. The widespread suppression of E2F-responsive genes, regardless of genetic heterogeneity among cell lines, suggests that the E2F transcriptional program is a primary target of BET inhibition in HPV-positive HNSCC. This effect is particularly significant given that HPV oncoproteins E6 and E7 typically lead to aberrant activation of E2F transcription factors and cell cycle dysregulation. By suppressing the E2F-dependent transcriptional program, JQ1 effectively counteracts these oncogenic effects of HPV. The downregulation of E2F target genes correlates strongly with the observed G1 cell cycle arrest in most cell lines, indicating a common mechanism of action. These findings align with observations in other cancer types, such as glioblastoma, suggesting a broadly applicable strategy in cancers with dysregulated cell cycle control (57). In HPV-positive HNSCC, the downregulation of E2F genes by JQ1 is particularly significant, as the HPV oncoproteins E6 and E7 are known to inactivate p53 and Rb, respectively, leading to the activation of E2F transcription factors and cell cycle dysregulation (49) on HPV oncoproteins and cell cycle regulation. The consistent suppression of E2F-responsive genes across cell lines could serve as a reliable biomarker of JQ1 activity and provides a strong rationale for combining BET inhibitors with therapies targeting cell cycle progression or DNA damage response pathways. While the exact mechanism may vary between cell lines, the consistency of this effect suggests a fundamental role for BET proteins in maintaining the E2F transcriptional program in HPV-positive HNSCC. This observation opens avenues for further research into the specific interactions between BET proteins, particularly BRD4, and E2F transcription factors in the context of HPV-driven oncogenesis.x Overall, our findings suggest that JQ1 has a dual role in inducing cell cycle arrest and p21-mediated cellular senescence, which could be exploited for therapeutic purposes. However, the resultant effects on genomic stability are not robust enough for clinical application as a standalone treatment. The therapeutic significance of our findings lies in the potential for combining BET inhibitors with standard radiation therapy in the treatment of HPV-positive HNSCC tumors. This combination approach could enhance the efficacy of radiation therapy by sensitizing cancer cells through the modulation of DDR and cell cycle regulation pathways. These findings highlight that JQ1’s effects are context-dependent and vary among different cell types and cancers and merit further investigation in HPV-positive HNSCC tumors.

## Conclusion

While our study provides valuable insights into the regulatory role of BET proteins and the potential therapeutic implications of JQ1 in HPV-associated HNSCC, it is important to acknowledge some limitations. First, our study was conducted using *in vitro* cell line models, which may not fully recapitulate the complexities of the tumor microenvironment and the heterogeneity of patient tumors. Further validation in patient-derived xenograft models or clinical samples would be necessary to confirm the observed effects and their relevance to human tumors. Second, the observed magnitude of gene expression changes to JQ1 treatment, particularly as quantified through cell cycle arrest and E2F target gene expression, highlights the need for further investigation to elucidate the underlying mechanisms driving these differences among cell lines. Third, while JQ1 is a potent BET inhibitor in the nanomolar range, second-generation BET inhibitors can provide more clinical relevance. Specific targeting of individual BET proteins or alternative BET inhibitors could help delineate the specific roles of BRD4 and other BET proteins for targeted therapies.

## Data Availability

The datasets presented in this study can be found in online repositories. The names of the repository/repositories and accession number(s) can be found in the article/**Supplementary Material**.
