# Supplementary material for: Targeted inhibition of BET proteins in HPV16-positive head and neck squamous cell carcinoma reveals heterogeneous transcriptional responses
Source: Front Oncol. 2024 Sep 5;14:1440836. doi: 10.3389/fonc.2024.1440836 (PMC11410754; doi:10.3389/fonc.2024.1440836)

Supplementary Figure. S1. (A) HPV-positive (UM-SCC-47, UD-SCC-2) head and neck cancer cell lines were treated with DMSO (vehicle), 0.5 µM (+)-JQ1 or (-)-JQ1 for 96 hours. The number of live cells in each condition was counted using a TC20 Biorad cell counter and trypan blue exclusion. The cell numbers were then normalized to the DMSO-treated group. With (+)-JQ1 treatment for 96 hours, the number of live cells was significantly reduced compared to vehicle control (DMSO). While the group treated with (-)-JQ1, a stereoisomer of (+)-JQ1, had a percentage of live cells similar to the vehicle control group. This suggests (-)-JQ1 has minimal effect on the proliferation of head and neck cancer cells at the concentration used in this study. **(B)** Cells were treated with 500 nM JQ1(+) or vehicle control JQ1(-) for 24 and 48 hours. Caspase 3/7 activity was quantified by luminescence and normalized to JQ1(-) treated controls. Data are presented as log2 fold change from three independent biological replicates.

A

B

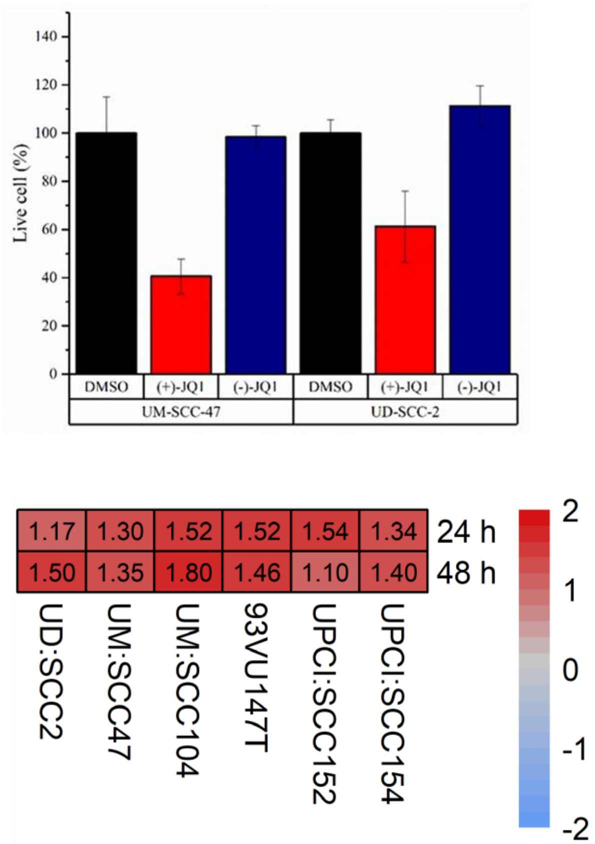

Supplement: Supplementary file 1 [file DataSheet1.pdf]
