# Supplementary material for: Targeted inhibition of BET proteins in HPV16-positive head and neck squamous cell carcinoma reveals heterogeneous transcriptional responses
Source: Front Oncol. 2024 Sep 5;14:1440836. doi: 10.3389/fonc.2024.1440836 (PMC11410754; doi:10.3389/fonc.2024.1440836)

Supplementary Figure S2. BRD4 knockdown phenocopies the effects of pan-BET inhibitor, JQ1, demonstrating downregulation of E2, E6, and E7 viral gene expression. **(A)** Stable BRD4 knockdown was assessed in the UM: SCC47 cell line by three independent shRNA clones, BRD4.1, 4.2, and 4.3, and quantified by qRT-PCR. Inset shows a western blot of Brd4 protein knockdown using anti-Brd4 antibodies. Knockdown efficiency is quantified above each bar. **(B, C)** Knockdown of BRD4 decreases E6, and E7 expression in UM: SCC47 cell line. Data represents the average ratio of viral gene expression relative to levels obtained from the control scrambled shRNA UMSSCC47 cell line.

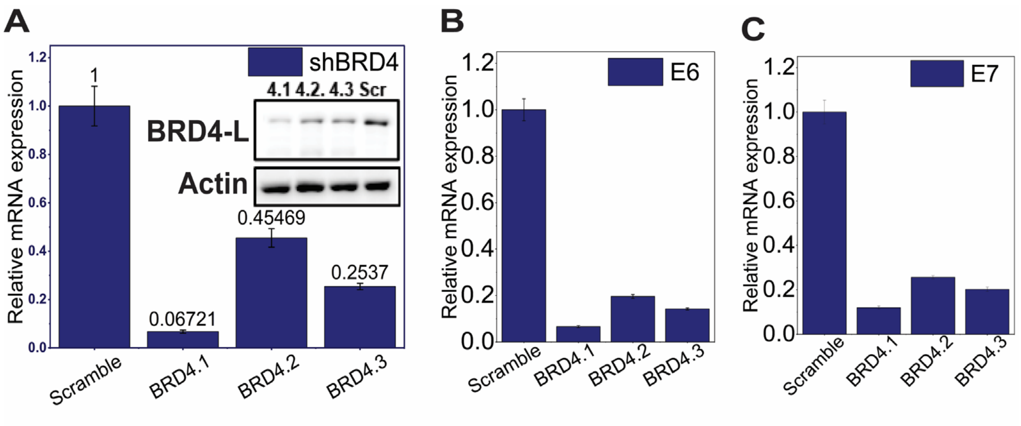

Supplement: Supplementary file 2 [file DataSheet2.pdf]
