# Supplementary material for: Targeted inhibition of BET proteins in HPV16-positive head and neck squamous cell carcinoma reveals heterogeneous transcriptional responses
Source: Front Oncol. 2024 Sep 5;14:1440836. doi: 10.3389/fonc.2024.1440836 (PMC11410754; doi:10.3389/fonc.2024.1440836)

Supplementary Figure S3. Western blot of HPV-negative (Tu-138, D562, FaDu) and positive (UD:SCC2 and UM:SCC47)head and neck cancer cells treated with JQ1 active and inactive enantiomers at 500 nM for 24 hours. The antibodies used are indicated in the methods section.

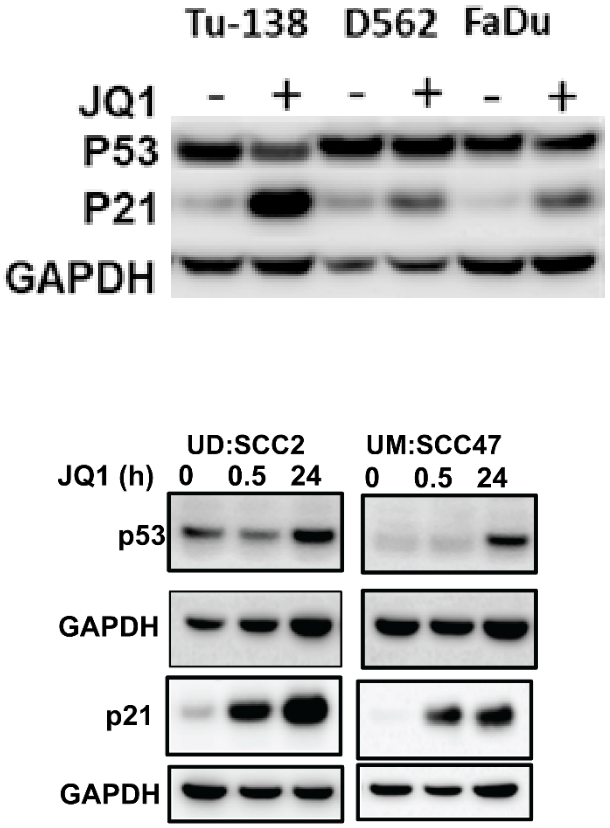

Supplement: Supplementary file 3 [file DataSheet3.pdf]
