# Supplementary material for: Targeted inhibition of BET proteins in HPV16-positive head and neck squamous cell carcinoma reveals heterogeneous transcriptional responses
Source: Front Oncol. 2024 Sep 5;14:1440836. doi: 10.3389/fonc.2024.1440836 (PMC11410754; doi:10.3389/fonc.2024.1440836)

Supplementary Table S1. The IC50 values for JQ1 treatments for all cell lines were extracted from applying the Nonlinear Curve Fit using the Levenberg Marquardt algorithm using the DoseResp model. The residual fits confirm that the IC50 values were fitted to the equation y = A1 + (A2-A1)/(1 + 10^((LOGx0-x)*p))

Nonlinear Curve Fit Dose Response Parameters

	Value	Standard Error	t-Value	Prob>|t|	Dependency
A1	42.49093	3.25572	13.05115	1.24718E-5	0.48754
A2	103.98268	13.99807	7.42836	3.06282E-4	0.84385
LOGx0	-7.18672	0.18486	-38.87645	1.93494E-8	0.83835
p	-1.66667	0.77999	-2.13678	0.07649	0.75364
span	61.49176	15.33673			
EC20	1.49458E-7	4.53401E-8			
EC50	6.50552E-8	2.76912E-8			
EC80	2.83169E-8	2.14424E-8			
A1	47.62577	2.86894	16.60048	3.04803E-6	0.43487
A2	98.32513	5.57133	17.64842	2.12477E-6	0.53333
LOGx0	-6.95525	0.12865	-54.06292	2.68886E-9	0.55615
p	-1.66667	0.65585	-2.54121	0.04401	0.53136
span	50.69936	6.73198			
EC20	2.54674E-7	8.61312E-8			
EC50	1.10853E-7	3.2838E-8			
EC80	4.82516E-8	2.53215E-8			
A1	52.08379	2.1801	23.89059	3.53199E-7	0.49136
A2	94.55697	4.12771	22.90787	4.5335E-7	0.61312
LOGx0	-6.79148	0.1189	-57.11942	1.93422E-9	0.5911
p	-1.66667	0.54071	-3.08235	0.0216	0.5907
span	42.47318	5.06964			
EC20	3.71323E-7	1.11008E-7			
EC50	1.61628E-7	4.42499E-8			
EC80	7.03525E-8	3.19445E-8			
A1	59.52425	1.43547	41.46682	1.31561E-8	0.28423
A2	97.63684	2.81272	34.71266	3.80814E-8	0.60298
LOGx0	-6.68143	0.08283	-80.66333	2.44453E-10	0.48042
p	-1.66667	0.47598	-3.50152	0.0128	0.4544
span	38.11259	3.35963			
EC20	4.78418E-7	1.28869E-7			
EC50	2.08244E-7	3.97174E-8			
EC80	9.06433E-8	3.04773E-8			
A1	16.85289	1.66196	10.14034	5.34922E-5	0.14153
A2	101.86837	15.80913	6.44364	6.61271E-4	0.69777
LOGx0	-7.0341	0.16566	-42.46106	1.14175E-8	0.75703
p	-1.66667	0.62101	-2.6838	0.03635	0.57388
span	85.01549	16.09625			
EC20	2.12393E-7	5.74289E-8			
EC50	9.24495E-8	3.52645E-8			
EC80	4.0241E-8	2.57667E-8			
A1	68.48517	4.95197	13.82989	8.8954E-6	0.40994
A2	109.4464	5.0427	21.70394	6.24656E-7	0.57057
LOGx0	-6.51796	0.28336	-23.00228	4.42407E-7	0.33373
p	-1.66667	1.578	-1.05619	0.33154	0.59263
span	40.96123	8.06861			
EC20	6.97064E-7	6.47238E-7			
EC50	3.03415E-7	1.97967E-7			
EC80	1.32069E-7	1.46452E-7			
A1	74.91165	8.45115	8.86407	1.14692E-4	0.80406
A2	107.50372	2.15514	49.88245	4.35383E-9	0.35344
LOGx0	-5.82047	0.22501	-25.86765	2.20081E-7	0.88767
p	-1.66667	1.24713	-1.3364	0.22986	0.77963
span	32.59208	9.01			
EC20	3.4735E-6	3.79727E-6			
EC50	1.51193E-6	7.83335E-7			
EC80	6.58104E-7	2.24638E-7			

Reduced Chi-sqr = 0.197218498215 COD(R^2) = 0.97049115006367
Iterations Performed = 12 Total Iterations in Session = 12
All datasets were fitted successfully.
Standard Error was scaled with square root of reduced Chi-Sqr. span, EC20, EC50, EC80 are derived parameter(s).

Statistics

	UM-SCC-47	UD-SCC-2	UPCI-SCC-90	93VU147T	UPCI-SCC-154	UM-SCC-104	UPCI-SCC-152
Number of Points	10	10	10	10	10	10	10
Degrees of Freedom	6	6	6	6	6	6	6
Reduced Chi-Sqr	0.69464	0.32555	0.31803	0.46139	1.30944	0.72104	0.19722
Residual Sum of Squares	4.16786	1.9533	1.90819	2.76832	7.85662	4.32623	1.18331
R-Square (COD)	0.96798	0.98163	0.95816	0.95835	0.98063	0.88452	0.97049
Adj. R-Square	0.95197	0.97245	0.93723	0.93753	0.97094	0.82678	0.95574
Fit Status	Succeeded(100)	Succeeded(100)	Succeeded(100)	Succeeded(100)	Succeeded(100)	Succeeded(100)	Succeeded(100)

Fit Status Code :
100 : Fit converged. Chi-Sqr tolerance value of 1E-9 was reached

		DF	Sum of Squares	Mean Square	F Value	Prob>F
	Regression	4	3505.71854	876.42963	1261.69645	6.69166E-9
UM-SCC-47	Residual	6	4.16786	0.69464		
	Uncorrected Total	10	3509.8864			
	Corrected Total	9	130.15589			
	Regression	4	1694.34777	423.58694	1301.14495	6.10211E-9
UD-SCC-2	Residual	6	1.9533	0.32555		
	Uncorrected Total	10	1696.30107			
	Corrected Total	9	106.33643			
	Regression	4	1764.8204	441.2051	1387.29699	5.03577E-9
UPCI-SCC-90	Residual	6	1.90819	0.31803		
	Uncorrected Total	10	1766.72859			
	Corrected Total	9	45.60214			
	Regression	4	1525.05995	381.26499	826.34737	2.37625E-8
93VU147T	Residual	6	2.76832	0.46139		
	Uncorrected Total	10	1527.82826			
	Corrected Total	9	66.4735			
	Regression	4	1350.58848	337.64712	257.85689	7.70464E-7
UPCI-SCC-154	Residual	6	7.85662	1.30944		
	Uncorrected Total	10	1358.4451			
	Corrected Total	9	405.51057			
	Regression	4	10685.41485	2671.35371	3704.87042	2.65067E-10
UM-SCC-104	Residual	6	4.32623	0.72104		
	Uncorrected Total	10	10689.74108			
	Corrected Total	9	37.4633			
	Regression	4	3210.35867	802.58967	4069.54558	2.0003E-10
UPCI-SCC-152	Residual	6	1.18331	0.19722		
	Uncorrected Total	10	3211.54198			
	Corrected Total	9	40.10021			

At the 0.05 level, the fitting function is significantly better than the function y=0.

Residual Plots

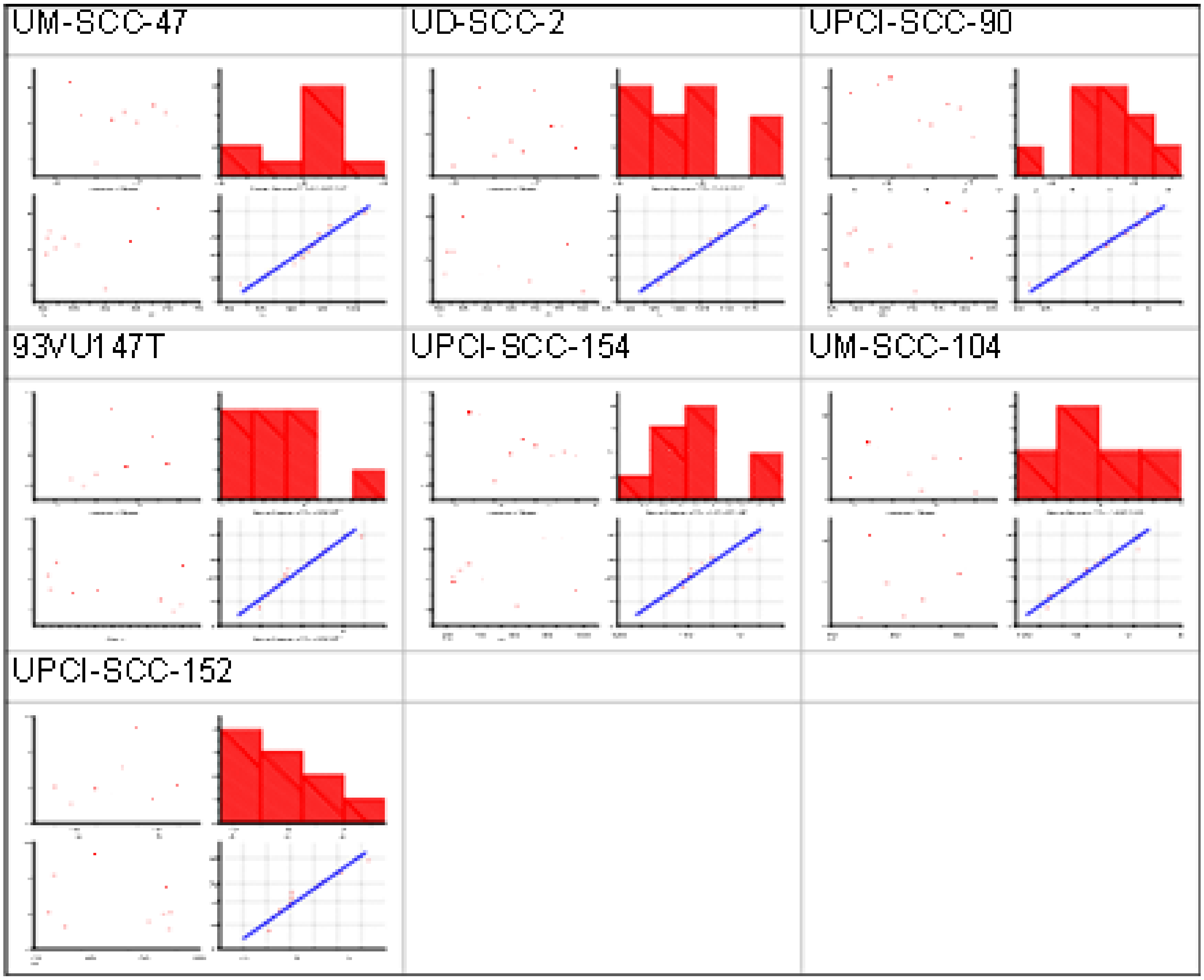

Supplement: Supplementary file 4 [file DataSheet4.pdf]
