# Supplementary material for: Targeted inhibition of BET proteins in HPV16-positive head and neck squamous cell carcinoma reveals heterogeneous transcriptional responses
Source: Front Oncol. 2024 Sep 5;14:1440836. doi: 10.3389/fonc.2024.1440836 (PMC11410754; doi:10.3389/fonc.2024.1440836)
Supplement: Supplementary file 5 [file DataSheet5.pdf]

Supplementary Table S2. The table shows the total number of reads per sample across different cell lines treated with JQ1 or untreated controls. Each row represents a sample, and the columns provide the following information: Sample Name: The name of the cell line sample, indicating the cell line (e.g., 93VU147T, UD-SCC2, UM-SCC47), treatment condition (JQ1 or control), and replicate number, total number of reads, mean coverage per sample, the min and max coverage depth for each sample . The percentage (%) of gene covered with minimum 10x: The percentage of the gene's length covered by at least 10 reads in the given sample. % of gene covered with minimum 50x: The percentage of the gene's length covered by at least 50 reads in the given sample. % of gene covered with minimum 100x: The percentage of the gene's length covered by at least 100 reads in the given sample. The table provides insights into the variability in coverage and depth across different genes, samples, and cell lines, which can be influenced by factors such as expression levels, viral copy numbers, and integration patterns.

Sample Nai	Total numb	Number of ı	Mean cover	Min coverag	Max covera	% of genom	% of genom	% of genome covered with minimum 100X
93VU147T_	32707294	8127	130.65	0	2227	46.60%	15.66%	12.99%
93VU147T_	53900710	11933	188.41	0	3213	56.29%	17.19%	13.51%
93VU147T_	15145914	3368	53.37	0	912	23.01%	12.75%	11.40%
93VU147T_	26157522	4263	68.11	0	1186	23.36%	12.71%	11.38%
93VU147T_	22277274	2538	40.99	0	650	28.71%	11.37%	9.69%
93VU147T_	30014290	5045	83.56	0	1342	60.42%	14.94%	10.08%
UD:SCC2_c	35070226	23455	333.85	4	3035	99.86%	91.89%	69.42%
UD:SCC2_c	35250846	17294	242.91	0	2524	98.31%	74.15%	53.88%
UD:SCC2_c	34692208	18841	267.08	10	2305	100.00%	84.27%	59.99%
UD:SCC2_J	39495356	10084	142.1	3	1281	98.37%	56.54%	34.92%
UD:SCC2_J	33712668	17828	252.8	1	2176	99.85%	80.55%	59.71%
UD:SCC2_J	33570648	19296	271.69	2	2346	99.85%	83.19%	62.42%
UM:SCC10	60580754	9489	163.7	0	1545	51.82%	48.01%	23.97%
UM:SCC10	54907674	8237	141.57	0	1360	51.26%	46.89%	21.84%
UM:SCC10	54842840	8180	140.8	0	1404	51.19%	45.93%	21.71%
UM:SCC10	54928308	1389	23.52	0	239	43.13%	10.31%	9.02%
UM:SCC10	37666692	900	15.56	0	161	35.16%	10.01%	5.41%
UM:SCC10	53824494	1210	20.68	0	198	41.68%	10.45%	8.63%
UM:SCC47	40588364	2200	32.15	0	474	20.33%	11.70%	9.20%
UM:SCC47	31001980	2225	35.88	0	505	25.25%	13.70%	10.13%
UM:SCC47	33965112	1733	25.42	0	378	18.87%	10.65%	8.08%
UM:SCC47	30776714	109	1.51	0	16	4.19%	0.00%	0.00%
UM:SCC47	31053250	136	1.88	0	26	6.35%	0.00%	0.00%
UM:SCC47	43732266	136	2.04	0	38	4.57%	0.00%	0.00%
UPCI:SCC1	33222650	12997	205.16	0	2536	89.69%	52.72%	34.24%
UPCI:SCC1	41776978	15725	249.64	0	3209	91.21%	51.34%	37.04%
UPCI:SCC1	36877594	14778	233.9	0	2763	94.78%	56.25%	41.66%
UPCI:SCC1	33155038	6707	106.43	0	1263	89.73%	41.44%	13.96%
UPCI:SCC1	30575022	7048	111.86	0	1491	87.90%	37.47%	15.00%
UPCI:SCC1	29930868	7793	123.94	0	2092	83.49%	35.62%	17.13%
UPCI:SCC1	57289924	5747	99.68	0	1741	23.80%	11.38%	9.75%
UPCI:SCC1	62851508	6053	108.18	0	1893	24.31%	11.56%	9.75%
UPCI:SCC1	58851012	6316	111.85	0	1921	24.06%	11.17%	9.78%
UPCI:SCC1	56442334	1747	29.83	0	572	17.51%	7.44%	7.37%
UPCI:SCC1	62912432	1853	31.91	0	575	21.92%	7.46%	7.36%
UPCI:SCC1	67275668	2064	37.04	0	636	23.36%	8.37%	7.35%
UPCI:SCC9	24452868	7329	119.95	0	1460	84.29%	30.10%	15.84%
UPCI:SCC9	30400904	9199	154.01	0	1861	93.89%	33.22%	23.54%
UPCI:SCC9	25946960	445	7.75	0	99	10.19%	6.32%	0.00%
UPCI:SCC9	14927068	552	9.16	0	72	20.59%	3.25%	0.00%
UPCI:SCC9	37315430	1848	29.47	0	218	72.70%	14.61%	8.35%
UPCI:SCC9	31931876	1358	20.86	0	170	54.22%	10.55%	5.97%
					95	14.44%	5.00%	0.00%
					69	16.32%	4.62%	0.00%
					25	3.17%	0.00%	0.00%
					10	0.67%	0.00%	0.00%
					10	0.10%	0.00%	0.00%
