# Supplementary material for: Targeted inhibition of BET proteins in HPV16-positive head and neck squamous cell carcinoma reveals heterogeneous transcriptional responses
Source: Front Oncol. 2024 Sep 5;14:1440836. doi: 10.3389/fonc.2024.1440836 (PMC11410754; doi:10.3389/fonc.2024.1440836)
Supplement: Supplementary file 6 [file DataSheet6.pdf]

Supplementary Table S3. The table shows the total number of HPV viral gene reads per sample across different cell lines treated with JQ1 or untreated controls. Each row represents a sample, and the columns provide the following information: Sample Name: The name of the cell line sample, indicating the cell line (e.g., 93VU147T, UD-SCC2, UM-SCC47), treatment condition (JQ1 or control), and replicate number, total number of reads, mean coverage per sample, the min and max coverage depth for each sample . The percentage (%) of gene covered with minimum 10x: The percentage of the gene's length covered by at least 10 reads in the given sample. % of gene covered with minimum 50x: The percentage of the gene's length covered by at least 50 reads in the given sample. % of gene covered with minimum 100x: The percentage of the gene's length covered by at least 100 reads in the given sample. The table provides insights into the variability in coverage and depth across different genes, samples, and cell lines, which can be influenced by factors such as expression levels, viral copy numbers, and integration patterns.

Sample Name	Gene Name	Mean coverage per gene	SD	% of gene covered with minimum 10x	% of gene covered with minimum 50x	% of gene covered with minimum 100x
93VU147T_JQ1_1	URR start	5.72	2.82	8.54	0	0
93VU147T_JQ1_1	E6	421.98	371.18	99.79	59.75	57.02
93VU147T_JQ1_1	E7	711.44	202.59	100	100	100
93VU147T_JQ1_1	E1	39.82	115.56	18.67	10.68	10.52
93VU147T_JQ1_1	E2	8.41	5.52	33.62	0	0
93VU147T_JQ1_1	E4	76.92	37.87	100	72.92	42.71
93VU147T_JQ1_1	E5	1.43	0.5	0	0	0
93VU147T_JQ1_1	L2	4.32	2.52	1.28	0	0
93VU147T_JQ1_1	L1	4.54	2.46	1.58	0	0
93VU147T_JQ1_1	URR end	2.44	1.52	0	0	0
93VU147T_JQ1_2	URR start	4.33	0.57	0	0	0
93VU147T_JQ1_2	E6	232.3	208.92	85.32	57.02	55.77
93VU147T_JQ1_2	E7	404.84	108.66	100	100	100
93VU147T_JQ1_2	E1	25.44	65.98	18.03	10.68	10.31
93VU147T_JQ1_2	E2	10.29	2.58	52.36	0	0
93VU147T_JQ1_2	E4	45.59	19.13	90.63	40.63	0
93VU147T_JQ1_2	E5	4.25	1.29	0	0	0
93VU147T_JQ1_2	L2	7.83	2.86	15.62	0	0

93VU147T_JQ1_2	L1	3.63	2.18	0	0	0
93VU147T_JQ1_2	URR end	2.4	1.36	0	0	0
93VU147T_JQ1_3	URR start	5.56	4.34	23.17	0	0
93VU147T_JQ1_3	E6	503.5	415.61	100	95.39	60.59
93VU147T_JQ1_3	E7	773.8	259.14	100	100	100
93VU147T_JQ1_3	E1	57.97	142.72	40.24	10.68	10.68
93VU147T_JQ1_3	E2	19.18	4.6	99.88	0	0
93VU147T_JQ1_3	E4	58.63	18.99	100	74.65	0
93VU147T_JQ1_3	E5	9.81	5.05	49.6	0	0
93VU147T_JQ1_3	L2	14.61	5.94	74.89	0	0
93VU147T_JQ1_3	L1	9.03	4.49	34.58	0	0
93VU147T_JQ1_3	URR end	5.35	2.36	0	0	0
93VU147T_control_1	URR start	14.68	8.55	57.32	0	0
93VU147T_control_1	E6	826.53	714.7	100	95.6	61.43
93VU147T_control_1	E7	1289.37	424.1	100	100	100
93VU147T_control_1	E1	73.47	194.32	51.82	10.68	10.68
93VU147T_control_1	E2	33.89	6.56	100	0	0
93VU147T_control_1	E4	175.32	81.58	100	94.44	78.82
93VU147T_control_1	E5	14.56	6.87	70.24	0	0
93VU147T_control_1	L2	7.39	3.06	15.34	0	0
93VU147T_control_1	L1	5.6	2.61	0.46	0	0
93VU147T_control_1	URR end	6.29	3.3	14.13	0	0
93VU147T_control_2	URR start	11.79	10.39	48.78	0	0
93VU147T_control_2	E6	1196.83	1052.69	100	96.23	68.76
93VU147T_control_2	E7	1884.35	578.33	100	100	100
93VU147T_control_2	E1	105.33	291.52	42.2	13.17	10.68

93VU147T_control_2	E2	31.34	10.03	100	6.7	0
93VU147T_control_2	E4	286.37	141.4	100	97.92	80.56
93VU147T_control_2	E5	8.78	3.94	25.79	0	0
93VU147T_control_2	L2	13.17	4.37	79.96	0	0
93VU147T_control_2	L1	7.21	3.16	20.29	0	0
93VU147T_control_2	URR end	4.48	2.17	0	0	0
93VU147T_control_3	URR start	4.2	4.16	7.32	0	0
93VU147T_control_3	E6	329.61	286.85	99.79	57.02	56.81
93VU147T_control_3	E7	536.93	153.92	100	100	100
93VU147T_control_3	E1	31.35	88.25	17.08	10.68	10.52
93VU147T_control_3	E2	8.96	4.08	31.39	0	0
93VU147T_control_3	E4	88.46	40.9	100	79.17	43.75
93VU147T_control_3	E5	0.96	0.99	0	0	0
93VU147T_control_3	L2	1.34	1.55	0	0	0
93VU147T_control_3	L1	3.53	1.78	0	0	0
93VU147T_control_3	URR end	1.01	1.1	0	0	0
UD:SCC2_JQ1_1	URR start	45.57	4.09	100	8.54	0
UD:SCC2_JQ1_1	E6	358.44	393.56	100	62.26	55.56
UD:SCC2_JQ1_1	E7	1039.32	105.31	100	100	100
UD:SCC2_JQ1_1	E1	51.65	95.33	99.95	26.65	3.33
UD:SCC2_JQ1_1	E2	193.06	119.51	100	76.55	70.22
UD:SCC2_JQ1_1	E4	261.79	83.52	100	100	91.32
UD:SCC2_JQ1_1	E5	206.92	80.3	100	100	92.46
UD:SCC2_JQ1_1	L2	83.72	22.78	100	97.36	22.61
UD:SCC2_JQ1_1	L1	76.78	60.07	100	50.72	47.96
UD:SCC2_JQ1_1	URR end	17.1	9.8	76.8	0	0

UD:SCC2_JQ1_2	URR start	101.83	11.68	100	100	54.88
UD:SCC2_JQ1_2	E6	688.03	692.07	100	100	91.82
UD:SCC2_JQ1_2	E7	1851.37	149.58	100	100	100
UD:SCC2_JQ1_2	E1	113.16	171.65	100	81.23	31.89
UD:SCC2_JQ1_2	E2	310.35	188.32	100	94.67	76.43
UD:SCC2_JQ1_2	E4	409.49	104.72	100	100	100
UD:SCC2_JQ1_2	E5	264.18	109.26	100	100	93.25
UD:SCC2_JQ1_2	L2	153.55	38.68	100	100	90.09
UD:SCC2_JQ1_2	L1	124.08	85.99	100	62.85	49.54
UD:SCC2_JQ1_2	URR end	52.95	33.85	100	26.27	20.53
UD:SCC2_JQ1_3	URR start	139.35	17.68	100	100	100
UD:SCC2_JQ1_3	E6	728.55	717.37	100	100	100
UD:SCC2_JQ1_3	E7	1912.15	185.56	100	100	100
UD:SCC2_JQ1_3	E1	121.29	183.05	100	89.64	28.45
UD:SCC2_JQ1_3	E2	355.1	201.91	100	100	86.72
UD:SCC2_JQ1_3	E4	426.99	108.37	100	100	100
UD:SCC2_JQ1_3	E5	294.74	115.65	100	100	94.05
UD:SCC2_JQ1_3	L2	158.16	37.47	100	100	95.72
UD:SCC2_JQ1_3	L1	144.25	104.7	100	52.77	51.12
UD:SCC2_JQ1_3	URR end	61.51	36.35	100	44.93	22.53
UD:SCC2_control_1	URR start	175.07	12.73	100	100	100
UD:SCC2_control_1	E6	972.56	971.9	100	100	100
UD:SCC2_control_1	E7	2451.17	227.41	100	100	100
UD:SCC2_control_1	E1	167.29	221.99	100	99.68	55.74
UD:SCC2_control_1	E2	408.59	239.93	100	100	80.15
UD:SCC2_control_1	E4	413.22	106.87	100	100	100

UD:SCC2_control_1	E5	350.85	163.46	100	100	93.65
UD:SCC2_control_1	L2	170.49	41.08	100	100	97.93
UD:SCC2_control_1	L1	169.07	113.94	100	85.31	51.91
UD:SCC2_control_1	URR end	78.45	58.49	100	50.4	26.27
UD:SCC2_control_2	URR start	94.41	15.54	100	100	48.78
UD:SCC2_control_2	E6	741.78	757.24	100	100	85.32
UD:SCC2_control_2	E7	2017.2	211.05	100	100	100
UD:SCC2_control_2	E1	103.27	178.32	100	68.64	25.97
UD:SCC2_control_2	E2	277.82	173.25	100	92.31	76.55
UD:SCC2_control_2	E4	389.45	100.63	100	100	100
UD:SCC2_control_2	E5	222.17	100.79	100	98.41	91.27
UD:SCC2_control_2	L2	117.59	24.51	100	99.43	75.11
UD:SCC2_control_2	L1	105.41	76.79	100	51.78	48.22
UD:SCC2_control_2	URR end	37.67	34.39	73.07	26.27	11.2
UD:SCC2_control_3	URR start	91.61	8.53	100	100	18.29
UD:SCC2_control_3	E6	759.95	747.88	100	100	100
UD:SCC2_control_3	E7	1992.98	144.42	100	100	100
UD:SCC2_control_3	E1	130.59	193.67	100	96.99	31.31
UD:SCC2_control_3	E2	320.14	200.35	100	99.75	74.57
UD:SCC2_control_3	E4	409.74	123.15	100	100	100
UD:SCC2_control_3	E5	289.95	101.61	100	100	93.65
UD:SCC2_control_3	L2	139.94	25.54	100	100	94.72
UD:SCC2_control_3	L1	126.79	86.58	100	57.44	50.59
UD:SCC2_control_3	URR end	55.41	30.32	100	29.6	15.87
UM:SCC104_JQ1_1	URR start	0.44	0.83	0	0	0
UM:SCC104_JQ1_1	E6	93.88	82.22	61.43	54.72	53.25

UM:SCC104_JQ1_1	E7	183.06	26.39	100	100	100
UM:SCC104_JQ1_1	E1	22.25	15.01	96.25	0.85	0.85
UM:SCC104_JQ1_1	E2	14.95	8.36	66.38	0	0
UM:SCC104_JQ1_1	E4	90.11	40.38	91.32	80.56	47.57
UM:SCC104_JQ1_1	E5	14.02	8.58	47.22	0	0
UM:SCC104_JQ1_1	L2	0.74	0.77	0	0	0
UM:SCC104_JQ1_1	L1	0.33	0.73	0	0	0
UM:SCC104_JQ1_1	URR end	0	0	0	0	0
UM:SCC104_JQ1_2	URR start	0	0	0	0	0
UM:SCC104_JQ1_2	E6	59.02	54.22	55.97	53.67	30.61
UM:SCC104_JQ1_2	E7	120.53	19.13	100	100	80.47
UM:SCC104_JQ1_2	E1	14.44	9.67	70.76	0.85	0.74
UM:SCC104_JQ1_2	E2	12.99	6.59	59.43	0	0
UM:SCC104_JQ1_2	E4	64.7	29.93	91.32	69.79	6.94
UM:SCC104_JQ1_2	E5	6.06	2.98	11.51	0	0
UM:SCC104_JQ1_2	L2	0	0	0	0	0
UM:SCC104_JQ1_2	L1	0.2	0.4	0	0	0
UM:SCC104_JQ1_2	URR end	0	0	0	0	0
UM:SCC104_JQ1_3	URR start	2.07	1.8	0	0	0
UM:SCC104_JQ1_3	E6	64.68	54.97	57.65	53.88	43.82
UM:SCC104_JQ1_3	E7	158.39	22.03	100	100	100
UM:SCC104_JQ1_3	E1	17.49	14.3	78.05	0.85	0.85
UM:SCC104_JQ1_3	E2	20.45	8.77	77.54	0	0
UM:SCC104_JQ1_3	E4	103.79	47.69	91.32	85.42	52.08
UM:SCC104_JQ1_3	E5	14.44	6.44	45.24	0	0
UM:SCC104_JQ1_3	L2	0.22	0.42	0	0	0

UM:SCC104_JQ1_3	L1	0.2	0.4	0	0	0
UM:SCC104_JQ1_3	URR end	0	0	0	0	0
UM:SCC104_control_1	URR start	11.34	9.37	57.32	0	0
UM:SCC104_control_1	E6	710.5	549.53	100	95.18	94.55
UM:SCC104_control_1	E7	1280.32	78.35	100	100	100
UM:SCC104_control_1	E1	96.28	116.56	100	98.47	20.41
UM:SCC104_control_1	E2	121.45	56.45	98.39	90.82	57.07
UM:SCC104_control_1	E4	910.26	410.82	100	100	91.32
UM:SCC104_control_1	E5	56.91	18.23	100	53.97	0
UM:SCC104_control_1	L2	1.97	1.51	0	0	0
UM:SCC104_control_1	L1	1.87	1.36	0	0	0
UM:SCC104_control_1	URR end	0.22	0.41	0	0	0
UM:SCC104_control_2	URR start	4.32	3.26	2.44	0	0
UM:SCC104_control_2	E6	599.3	478	100	95.18	70.65
UM:SCC104_control_2	E7	1136.8	71.83	100	100	100
UM:SCC104_control_2	E1	82.8	96.55	100	93.44	18.93
UM:SCC104_control_2	E2	100.67	38.15	98.39	89.33	53.85
UM:SCC104_control_2	E4	788.9	336.44	100	100	91.32
UM:SCC104_control_2	E5	60.12	24.5	100	55.56	5.95
UM:SCC104_control_2	L2	1.79	1.26	0	0	0
UM:SCC104_control_2	L1	1.45	1.29	0	0	0
UM:SCC104_control_2	URR end	0.29	0.48	0	0	0
UM:SCC104_control_3	URR start	2.83	3.57	0	0	0
UM:SCC104_control_3	E6	612.32	495.88	98.53	95.18	74.21
UM:SCC104_control_3	E7	1110.06	95.54	100	100	100
UM:SCC104_control_3	E1	93.61	91.59	100	99.95	20.73

UM:SCC104_control_3	E2	86.85	37.55	98.39	76.8	45.91
UM:SCC104_control_3	E4	751.97	328.49	100	91.32	91.32
UM:SCC104_control_3	E5	42.44	23.47	100	34.92	0
UM:SCC104_control_3	L2	0.83	0.86	0	0	0
UM:SCC104_control_3	L1	3.43	2.25	0	0	0
UM:SCC104_control_3	URR end	1.03	1.11	0	0	0
UM:SCC47_JQ1_1	URR start	1	0	0	0	0
UM:SCC47_JQ1_1	E6	4.02	3.53	2.31	0	0
UM:SCC47_JQ1_1	E7	10.63	2.55	70.71	0	0
UM:SCC47_JQ1_1	E1	1.11	1.6	0	0	0
UM:SCC47_JQ1_1	E2	1.16	1.54	0	0	0
UM:SCC47_JQ1_1	E4	0.47	0.5	0	0	0
UM:SCC47_JQ1_1	E5	6.33	3.84	0	0	0
UM:SCC47_JQ1_1	L2	0.21	0.61	0	0	0
UM:SCC47_JQ1_1	L1	1.03	1.16	0	0	0
UM:SCC47_JQ1_1	URR end	0.09	0.29	0	0	0
UM:SCC47_JQ1_2	URR start	3.11	1.67	0	0	0
UM:SCC47_JQ1_2	E6	9.52	6.95	51.99	0	0
UM:SCC47_JQ1_2	E7	17.55	5.34	82.15	0	0
UM:SCC47_JQ1_2	E1	0.71	1.3	0	0	0
UM:SCC47_JQ1_2	E2	1.72	1.59	0	0	0
UM:SCC47_JQ1_2	E4	0.81	0.4	0	0	0
UM:SCC47_JQ1_2	E5	4.19	1.79	0	0	0
UM:SCC47_JQ1_2	L2	0.24	0.43	0	0	0
UM:SCC47_JQ1_2	L1	0.21	0.41	0	0	0
UM:SCC47_JQ1_2	URR end	0	0	0	0	0

UM:SCC47_JQ1_3	URR start	0.02	0.16	0	0	0
UM:SCC47_JQ1_3	E6	4.14	3.44	9.64	0	0
UM:SCC47_JQ1_3	E7	24.29	7.93	100	0	0
UM:SCC47_JQ1_3	E1	2.05	2.12	0	0	0
UM:SCC47_JQ1_3	E2	2.09	1.54	0	0	0
UM:SCC47_JQ1_3	E4	0.45	0.5	0	0	0
UM:SCC47_JQ1_3	E5	1.91	1.27	0	0	0
UM:SCC47_JQ1_3	L2	0	0	0	0	0
UM:SCC47_JQ1_3	L1	0.27	0.44	0	0	0
UM:SCC47_JQ1_3	URR end	0.4	0.49	0	0	0
UM:SCC47_control_1	URR start	0.9	1.14	0	0	0
UM:SCC47_control_1	E6	187.18	152	96.23	67.51	53.25
UM:SCC47_control_1	E7	364.27	91.02	100	100	100
UM:SCC47_control_1	E1	7.41	7.63	11.85	0.85	0.11
UM:SCC47_control_1	E2	19.01	37.79	18.98	13.77	7.57
UM:SCC47_control_1	E4	0	0	0	0	0
UM:SCC47_control_1	E5	88.09	49.32	100	66.67	40.87
UM:SCC47_control_1	L2	1.07	1.06	0	0	0
UM:SCC47_control_1	L1	0.72	1.36	0	0	0
UM:SCC47_control_1	URR end	0.39	0.49	0	0	0
UM:SCC47_control_2	URR start	11.55	1.96	58.54	0	0
UM:SCC47_control_2	E6	194	143.01	100	85.74	54.72
UM:SCC47_control_2	E7	395.1	82.12	100	100	100
UM:SCC47_control_2	E1	8.7	9.27	18.98	0.85	0.32
UM:SCC47_control_2	E2	22.83	43.33	31.27	14.02	8.81
UM:SCC47_control_2	E4	2.02	0.81	0	0	0

UM:SCC47_control_2	E5	119.98	53.13	100	92.46	62.3
UM:SCC47_control_2	L2	1.12	1.53	0	0	0
UM:SCC47_control_2	L1	1.51	2.02	0	0	0
UM:SCC47_control_2	URR end	0.5	1.38	0	0	0
UM:SCC47_control_3	URR start	3.98	2.45	2.44	0	0
UM:SCC47_control_3	E6	147.94	118.46	99.79	55.14	51.15
UM:SCC47_control_3	E7	288.92	77.35	100	100	98.99
UM:SCC47_control_3	E1	5.76	5.73	11.05	0.48	0
UM:SCC47_control_3	E2	14.28	27.6	15.51	11.54	2.11
UM:SCC47_control_3	E4	0.63	0.48	0	0	0
UM:SCC47_control_3	E5	69.83	35.83	100	65.48	30.56
UM:SCC47_control_3	L2	0.59	0.93	0	0	0
UM:SCC47_control_3	L1	0.66	0.61	0	0	0
UM:SCC47_control_3	URR end	0.68	1.13	0	0	0
UPCI:SCC152_JQ1_1	URR start	56.44	6.41	100	79.27	0
UPCI:SCC152_JQ1_1	E6	591.36	470.47	100	100	62.47
UPCI:SCC152_JQ1_1	E7	816.86	224.08	100	100	100
UPCI:SCC152_JQ1_1	E1	58.62	30.26	100	61.08	1.06
UPCI:SCC152_JQ1_1	E2	80.88	24.37	100	95.78	21.09
UPCI:SCC152_JQ1_1	E4	169.83	38.6	100	100	91.32
UPCI:SCC152_JQ1_1	E5	66.57	24.71	100	58.33	15.08
UPCI:SCC152_JQ1_1	L2	14.81	4.63	77.39	0	0
UPCI:SCC152_JQ1_1	L1	17.84	5.37	89.92	0	0
UPCI:SCC152_JQ1_1	URR end	18.84	18.2	43.73	4.53	0
UPCI:SCC152_JQ1_2	URR start	63.46	10.82	100	96.34	0
UPCI:SCC152_JQ1_2	E6	645.38	539.78	100	99.79	57.23

UPCI:SCC152_JQ1_2	E7	879.99	342.11	100	100	100
UPCI:SCC152_JQ1_2	E1	61.56	40.06	99.84	55	8.46
UPCI:SCC152_JQ1_2	E2	71.8	25.13	100	81.76	19.73
UPCI:SCC152_JQ1_2	E4	203.82	54.19	100	100	91.32
UPCI:SCC152_JQ1_2	E5	41.87	17.6	100	17.06	0
UPCI:SCC152_JQ1_2	L2	12.44	5.19	58.84	0	0
UPCI:SCC152_JQ1_2	L1	18.62	4.68	95.06	0	0
UPCI:SCC152_JQ1_2	URR end	16.83	16.84	40.4	4.53	0
UPCI:SCC152_JQ1_3	URR start	63.29	4.14	100	100	0
UPCI:SCC152_JQ1_3	E6	803.26	700.61	100	98.32	67.51
UPCI:SCC152_JQ1_3	E7	985.97	598.96	100	100	100
UPCI:SCC152_JQ1_3	E1	70.51	48.47	99.68	61.13	24.91
UPCI:SCC152_JQ1_3	E2	54.34	13.25	100	52.23	0
UPCI:SCC152_JQ1_3	E4	185.81	58.13	100	100	86.81
UPCI:SCC152_JQ1_3	E5	27.33	6.85	100	0	0
UPCI:SCC152_JQ1_3	L2	10.5	4.04	49.07	0	0
UPCI:SCC152_JQ1_3	L1	17.09	8.06	78.26	0	0
UPCI:SCC152_JQ1_3	URR end	14.6	16.5	40.13	6.27	0
UPCI:SCC152_control_1	URR start	109.99	9.83	100	100	76.83
UPCI:SCC152_control_1	E6	1190.32	924.77	100	100	100
UPCI:SCC152_control_1	E7	1806.31	288.88	100	100	100
UPCI:SCC152_control_1	E1	115.7	84.31	100	85.67	57.38
UPCI:SCC152_control_1	E2	123.5	62.17	100	100	35.11
UPCI:SCC152_control_1	E4	272.08	66.5	100	100	91.32
UPCI:SCC152_control_1	E5	128.51	42.81	100	100	68.65
UPCI:SCC152_control_1	L2	13.81	3.83	80.31	0	0

UPCI:SCC152_control_1	L1	14.14	4.35	78.85	0	0
UPCI:SCC152_control_1	URR end	31.4	34.72	49.73	33.6	2.13
UPCI:SCC152_control_2	URR start	118.26	19.71	100	100	67.07
UPCI:SCC152_control_2	E6	1483.4	1157.87	100	100	100
UPCI:SCC152_control_2	E7	2275.65	419.79	100	100	100
UPCI:SCC152_control_2	E1	124.37	95.28	100	79.59	59.33
UPCI:SCC152_control_2	E2	159.66	100.34	100	100	54.22
UPCI:SCC152_control_2	E4	313.97	77.31	100	100	91.32
UPCI:SCC152_control_2	E5	159.66	67.72	100	100	89.68
UPCI:SCC152_control_2	L2	14.73	4.88	73.75	0	0
UPCI:SCC152_control_2	L1	16.37	4.7	86.43	0	0
UPCI:SCC152_control_2	URR end	27.97	29.26	50.13	32.13	0
UPCI:SCC152_control_3	URR start	112.51	9.76	100	100	91.46
UPCI:SCC152_control_3	E6	1300.7	988.73	100	100	100
UPCI:SCC152_control_3	E7	1942.84	354.53	100	100	100
UPCI:SCC152_control_3	E1	147.34	91.34	100	99.52	61.71
UPCI:SCC152_control_3	E2	170.7	91.56	100	100	92.93
UPCI:SCC152_control_3	E4	312.59	75.81	100	100	98.61
UPCI:SCC152_control_3	E5	156.07	76.06	100	100	76.59
UPCI:SCC152_control_3	L2	16.13	4.32	90.01	0	0
UPCI:SCC152_control_3	L1	19.18	7.11	87.94	0	0
UPCI:SCC152_control_3	URR end	36.99	35.1	73.33	38.27	1.2
UPCI:SCC154_JQ1_1	URR start	0	0	0	0	0
UPCI:SCC154_JQ1_1	E6	240.34	213.56	92.24	55.97	54.93
UPCI:SCC154_JQ1_1	E7	352.97	87.94	100	100	100
UPCI:SCC154_JQ1_1	E1	7.55	12.52	32.31	0.85	0.85

UPCI:SCC154_JQ1_1	E2	0.23	0.5	0	0	0
UPCI:SCC154_JQ1_1	E4	0	0	0	0	0
UPCI:SCC154_JQ1_1	E5	0.38	0.68	0	0	0
UPCI:SCC154_JQ1_1	L2	0.01	0.08	0	0	0
UPCI:SCC154_JQ1_1	L1	0	0	0	0	0
UPCI:SCC154_JQ1_1	URR end	0	0	0	0	0
UPCI:SCC154_JQ1_2	URR start	0	0	0	0	0
UPCI:SCC154_JQ1_2	E6	240.86	209.45	95.39	56.18	55.14
UPCI:SCC154_JQ1_2	E7	374.18	92.74	100	100	100
UPCI:SCC154_JQ1_2	E1	12.67	16.07	48.7	0.85	0.74
UPCI:SCC154_JQ1_2	E2	0.67	1.24	0	0	0
UPCI:SCC154_JQ1_2	E4	0	0	0	0	0
UPCI:SCC154_JQ1_2	E5	0	0	0	0	0
UPCI:SCC154_JQ1_2	L2	0	0	0	0	0
UPCI:SCC154_JQ1_2	L1	0	0	0	0	0
UPCI:SCC154_JQ1_2	URR end	0	0	0	0	0
UPCI:SCC154_JQ1_3	URR start	1.15	1	0	0	0
UPCI:SCC154_JQ1_3	E6	271.17	223.59	95.18	71.49	55.14
UPCI:SCC154_JQ1_3	E7	456.3	119.75	100	100	100
UPCI:SCC154_JQ1_3	E1	13.49	16.41	57.11	0.85	0.69
UPCI:SCC154_JQ1_3	E2	0	0	0	0	0
UPCI:SCC154_JQ1_3	E4	0.48	0.5	0	0	0
UPCI:SCC154_JQ1_3	E5	0	0	0	0	0
UPCI:SCC154_JQ1_3	L2	0	0	0	0	0
UPCI:SCC154_JQ1_3	L1	0	0	0	0	0
UPCI:SCC154_JQ1_3	URR end	0	0	0	0	0

UPCI:SCC154_control_1	URR start	1.79	2.07	0	0	0
UPCI:SCC154_control_1	E6	765.29	573.37	97.06	94.97	94.34
UPCI:SCC154_control_1	E7	1248.06	275.91	100	100	100
UPCI:SCC154_control_1	E1	24.32	39.39	58.86	7.09	0.85
UPCI:SCC154_control_1	E2	0.06	0.55	0	0	0
UPCI:SCC154_control_1	E4	0	0	0	0	0
UPCI:SCC154_control_1	E5	0	0	0	0	0
UPCI:SCC154_control_1	L2	0	0	0	0	0
UPCI:SCC154_control_1	L1	0	0	0	0	0
UPCI:SCC154_control_1	URR end	0	0	0	0	0
UPCI:SCC154_control_2	URR start	5.94	3.36	19.51	0	0
UPCI:SCC154_control_2	E6	801.01	609.77	100	95.18	94.34
UPCI:SCC154_control_2	E7	1401.33	359.5	100	100	100
UPCI:SCC154_control_2	E1	25.93	42.89	59.44	6.56	0.85
UPCI:SCC154_control_2	E2	0.01	0.11	0	0	0
UPCI:SCC154_control_2	E4	0	0	0	0	0
UPCI:SCC154_control_2	E5	0.03	0.18	0	0	0
UPCI:SCC154_control_2	L2	0	0	0	0	0
UPCI:SCC154_control_2	L1	0	0	0	0	0
UPCI:SCC154_control_2	URR end	0.69	1.26	0	0	0
UPCI:SCC154_control_3	URR start	3.23	3.49	0	0	0
UPCI:SCC154_control_3	E6	842.37	644.55	99.58	95.18	94.76
UPCI:SCC154_control_3	E7	1444.46	348.94	100	100	100
UPCI:SCC154_control_3	E1	23.9	42.72	58.7	5.02	0.85
UPCI:SCC154_control_3	E2	0.85	1.35	0	0	0
UPCI:SCC154_control_3	E4	0.13	0.6	0	0	0

UPCI:SCC154_control_3	E5	0.4	1.03	0	0	0
UPCI:SCC154_control_3	L2	0.03	0.24	0	0	0
UPCI:SCC154_control_3	L1	0.01	0.13	0	0	0
UPCI:SCC154_control_3	URR end	0	0	0	0	0
UPCI:SCC90_JQ1_1	URR start	3.45	0.59	0	0	0
UPCI:SCC90_JQ1_1	E6	24.18	19.99	55.14	7.13	0
UPCI:SCC90_JQ1_1	E7	54.49	8.64	100	63.97	0
UPCI:SCC90_JQ1_1	E1	5.39	4.55	3.91	0	0
UPCI:SCC90_JQ1_1	E2	8.73	4.06	41.32	0	0
UPCI:SCC90_JQ1_1	E4	30.71	10.97	91.32	0	0
UPCI:SCC90_JQ1_1	E5	8.22	3.77	35.32	0	0
UPCI:SCC90_JQ1_1	L2	2.72	1.75	0	0	0
UPCI:SCC90_JQ1_1	L1	6.51	2.58	6.06	0	0
UPCI:SCC90_JQ1_1	URR end	2.11	2.11	0	0	0
UPCI:SCC90_JQ1_2	URR start	5.68	1.85	0	0	0
UPCI:SCC90_JQ1_2	E6	82.82	66	98.95	51.78	49.27
UPCI:SCC90_JQ1_2	E7	176.13	18.25	100	100	100
UPCI:SCC90_JQ1_2	E1	19.17	12.46	80.43	0.85	0.85
UPCI:SCC90_JQ1_2	E2	29.91	22.51	87.97	28.41	0
UPCI:SCC90_JQ1_2	E4	88.82	22.26	100	91.32	34.03
UPCI:SCC90_JQ1_2	E5	40.13	14	100	35.32	0
UPCI:SCC90_JQ1_2	L2	8.72	3.56	28.39	0	0
UPCI:SCC90_JQ1_2	L1	17.71	4.43	93.94	0	0
UPCI:SCC90_JQ1_2	URR end	3.86	2.26	0	0	0
UPCI:SCC90_JQ1_3	URR start	6.32	0.99	0	0	0
UPCI:SCC90_JQ1_3	E6	64.57	52.58	86.58	51.57	33.54

UPCI:SCC90_ JQ1_3	E7	138.78	16.6 8	100	100	100
UPCI:SCC90_ JQ1_3	E1	12.15	9.08	50.03	0.85	0.05
UPCI:SCC90_ JQ1_3	E2	16.47	15.1 1	38.83	0.99	0
UPCI:SCC90_ JQ1_3	E4	55.74	16.6 4	99.65	76.74	0
UPCI:SCC90_ JQ1_3	E5	29.89	10.9 2	100	5.16	0
UPCI:SCC90_ JQ1_3	L2	7.23	3.96	25.32	0	0
UPCI:SCC90_ JQ1_3	L1	11	2.59	55.47	0	0
UPCI:SCC90_ JQ1_3	URR end	4.74	3.26	1.47	0	0
UPCI:SCC90_ control_1	URR start	20.05	8.01	95.12	0	0
UPCI:SCC90_ control_1	E6	599.63	519. 67	100	96.65	56.18
UPCI:SCC90_ control_1	E7	1166.82	166. 22	100	100	100
UPCI:SCC90_ control_1	E1	53.77	58.4 4	98.78	46.11	2.8
UPCI:SCC90_ control_1	E2	63.06	62.9 7	100	28.41	28.41
UPCI:SCC90_ control_1	E4	227.33	67.5 9	100	91.32	91.32
UPCI:SCC90_ control_1	E5	108.08	46.0 2	100	92.06	47.62
UPCI:SCC90_ control_1	L2	12.25	6.9	52.57	0	0
UPCI:SCC90_ control_1	L1	23.56	7	98.29	0	0
UPCI:SCC90_ control_1	URR end	7.02	4.62	21.6	0	0
UPCI:SCC90_ control_2	URR start	17.4	4.82	95.12	0	0
UPCI:SCC90_ control_2	E6	729.88	630. 26	100	96.23	60.8
UPCI:SCC90_ control_2	E7	1546.36	205. 09	100	100	100
UPCI:SCC90_ control_2	E1	74.75	73.8 3	100	54.84	33.53
UPCI:SCC90_ control_2	E2	85.45	79.9 8	100	28.41	28.41
UPCI:SCC90_ control_2	E4	236.13	62.9 4	100	91.32	91.32
UPCI:SCC90_ control_2	E5	115.38	55.2 5	100	99.6	42.46
UPCI:SCC90_ control_2	L2	19.4	6.64	92.58	0	0

UPCI:SCC90_ control_2	L1	35.33	7.08	100	2.7	0
UPCI:SCC90_ control_2	URR end	8.65	6.28	27.73	0	0
UPCI:SCC90_ control_3	URR start	0	0	0	0	0
UPCI:SCC90_ control_3	E6	35.14	31.5 4	54.93	48.22	0
UPCI:SCC90_ control_3	E7	76.99	15.7 3	100	87.88	0
UPCI:SCC90_ control_3	E1	4.87	3.7	6.72	0	0
UPCI:SCC90_ control_3	E2	3.39	2.7	0	0	0
UPCI:SCC90_ control_3	E4	7.3	2.37	0	0	0
UPCI:SCC90_ control_3	E5	3.88	1.93	0	0	0
UPCI:SCC90_ control_3	L2	1.43	1.01	0	0	0
UPCI:SCC90_ control_3	L1	2.12	1.33	0	0	0
UPCI:SCC90_ control_3	URR end	0.71	0.79	0	0	0
