# Supplementary material for: Targeted inhibition of BET proteins in HPV16-positive head and neck squamous cell carcinoma reveals heterogeneous transcriptional responses
Source: Front Oncol. 2024 Sep 5;14:1440836. doi: 10.3389/fonc.2024.1440836 (PMC11410754; doi:10.3389/fonc.2024.1440836)
Supplement: Supplementary file 7 [file DataSheet7.pdf]

Supplementary Table S4

| Gene | logfc (SCC | logfc (SCC | logfc (SCC | logfc (SCC | logfc (SCC | logfc (SCC | logfc (SCC154 |
|---|---|---|---|---|---|---|---|
| ADA | -1.418 | -0.329 | 1.216473 | -1.40306 | -0.741 | -2.997 | -1.84048 |
| CCNE1 | 0.491 | -1.85257 | -1.633 | -0.655 | -0.424 | 0.217 | -0.105 |
| CRIP2 | 0.161 | -1.16426 | 1.621 | -1.14874 | -0.012 | -0.236 | -1.72665 |
| DNA2 | -0.405 | -2.28346 | -0.86 | -1.15021 | -0.541 | -0.336 | -0.837 |
| DNMT1 | 0.361 | -1.10659 | -0.323 | -0.45 | -0.188 | 0.005 | -0.137 |
| EPHX1 | 0.842 | 1.205612 | 1.146 | 1.312281 | 0.212 | 0.642 | 1.707048 |
| GANC | -1.807 | -2.02064 | -0.527 | -1.09279 | -0.625 | -0.23 | -1.38736 |
| GRB7 | -0.315 | 0.012 | 2.054 | 1.23153 | 0.11 | 0.819 | 1.527069 |
| MSH6 | 0.007 | -1.2066 | -1.015 | -1.207 | -0.046 | -0.091 | -0.394 |
| STMN1 | 0.353 | -1.29946 | 0.099 | 0.14 | -0.28 | -0.231 | -0.102 |
| MCM6 | -0.175 | -2.16718 | -0.813 | -0.358 | -0.046 | -0.242 | -0.274 |
| MCM7 | -0.118 | -2.38168 | -0.976 | -0.709 | -0.494 | -0.589 | -0.648 |
| MDM2 | 0.099 | 1.696673 | 1.96 | 2.483156 | 0.257 | 1.683 | 1.187127 |
| MRE11A | -0.229 | -0.75615 | -0.746 | -0.12 | 0.005 | 0.13 | -0.149 |
| MYC | -0.639 | -0.66751 | -3.654 | -1.35065 | -0.424 | -0.895 | -1.33402 |
| NME4 | -1.96 | -1.49969 | -1.61713 | -1.39219 | -1.044 | -0.23 | -2.427 |
| PCNA | 0.403 | -1.40063 | -0.157 | 0.101 | -0.291 | -0.026 | -0.196 |
| POLE2 | -1.145 | -3.40857 | -1.997 | -2.13605 | -0.638 | -1.131 | -1.8334 |
| PRIM1 | -0.028 | -2.69714 | -1.537 | -1.22317 | -0.423 | -0.455 | -0.956 |
| MAPK13 | -3.155 | -0.8506 | -0.435 | -2.03413 | -0.245 | -1.294 | -1.62937 |
| RFC3 | -0.284 | -2.06199 | -1.796 | -1.30253 | -0.708 | -0.647 | -1.04719 |
| RPA1 | 0.284 | -0.77616 | -0.234 | -0.197 | -0.081 | -0.297 | -0.109 |
| RRM2 | 0.475 | -3.657 | -1.145 | -0.75 | -0.696 | -0.182 | -0.313 |
| SFRP1 | 1.754 | 1.552295 | 1.259496 | -0.53611 | -0.113 | 0.678 | 1.109763 |
| ST3GAL4 | -0.735 | -0.532 | 0.788 | -0.79 | -0.339 | -0.091 | -1.16151 |
| SMARCA1 | -0.092 | -0.274 | -2.049 | -1.52009 | 0.124 | 0.036 | -2.2446 |
| SPOCK1 | 0.833 | -0.044 | -1.934 | -0.248 | -0.31 | -1.291 | -1.82271 |
| TGM1 | -4.821 | -1.89315 | 0.183 | -1.66145 | -1.3197 | -1.106 | -1.45082 |
| THRA | -2.051 | -1.02599 | -1.428 | -1.35209 | -0.468 | -0.068 | -0.934 |
| BTG2 | 0.699 | 0.187 | 1.621 | 1.46333 | 0.166 | 1.302 | 0.588 |
| ACOX3 | 0.113 | 0.708818 | 0.494 | 0.579 | -0.367 | -0.272 | 0.339 |
| PLA2G6 | -0.068 | 0.015 | -0.284 | 1.106864 | 0.026 | -0.071 | 1.257278 |
| RAD54L | 0.088 | -3.44377 | -0.998 | -1.6597 | -0.943 | -0.926 | -1.28743 |
| TNFSF9 | 1.332 | 0.832053 | 2.324 | 2.541196 | 0.391 | 0.148 | 1.80766 |
| TIMELESS | 0.403 | -1.17628 | -0.355 | 0.214 | -0.12 | 0.083 | -0.073 |
| BAZ1B | -0.21 | -0.74366 | -0.556 | -0.41 | -0.098 | -0.012 | -0.484 |
| ARTN | -1.902 | -0.531 | -0.066 | -1.69475 | -0.537 | -1.332 | -1.26554 |
| SLC7A7 | -1.081 | -1.8849 | | -1.47004 | 0.208033 | 0.365729 | -0.668 |
| CCNE2 | 0.222 | -2.15843 | -0.742 | -0.861 | -0.197 | 0.2 | -0.212 |
| ZW10 | -0.2 | -0.64412 | -0.729 | -0.479 | -0.091 | -0.148 | -0.505 |
| IER2 | 0.962 | 0.726338 | 2.086 | 0.741 | 0.046 | 0.447 | 0.672 |
| SIVA1 | -0.032 | -1.1962 | 0.126 | -1.01091 | -0.438 | -1.281 | -0.566 |
| MAN1B1 | 0.338 | 0.675238 | 1.354 | 0.785 | 0.247 | 0.209 | 1.150386 |

| | | | | | | | |
|---|---|---|---|---|---|---|---|
| PLEKHA6 | 0.581 | -0.60938 | -1.078 | -0.295 | 0.477 | -1.233 | 0.162 |
| DNAJC9 | 0.425 | -1.00536 | -0.592 | -0.451 | -0.118 | -0.14 | -0.221 |
| HEY2 | 0.56 | 1.677 | -0.357 | 2.814 | 0.275779 | 0.293 | 2.285511 |
| IL36RN | -2.72361 | | -3.535 | -4.42385 | -0.99665 | -1.253 | -6.7151 |
| BBC3 | -0.069 | 1.418772 | 1.547 | 1.848454 | 1.574 | 0.118 | 1.906123 |
| UBE2T | 0.309 | -1.87799 | -0.841 | -1.09706 | -0.658 | -0.631 | -0.698 |
| NXT1 | -0.452 | -0.91789 | 0.506 | -1.33701 | -0.305 | -0.814 | -1.15179 |
| SLC2A8 | 1.115 | 0.898389 | 0.506 | 0.139 | -0.183 | -0.548 | 0.898 |
| DCXR | 2.261 | 2.005127 | 2.043 | 2.434872 | 0.61 | 1.427 | 1.669046 |
| GNB1L | 0.617 | -0.088 | 0.128 | 1.032582 | -0.334 | -0.537 | 0.357 |
| MANSC1 | -1.291 | -1.30386 | -0.224 | -0.932 | -0.64357 | 0.031 | -1.46512 |
| PALMD | -1.566 | -0.285 | -0.812 | -3.85019 | -0.775 | -1.191 | |
| TIPIN | -0.603 | -1.24734 | -1.635 | -0.688 | -0.578 | -0.434 | -1.10036 |
| CLN6 | -0.185 | -0.86289 | -0.683 | -0.972 | -0.394 | -0.529 | -0.472 |
| SULF2 | 0.256 | 0.155 | -0.314 | -0.269 | 0.187 | 1.258 | -1.15602 |
| SLC2A9 | -2.625 | -0.98809 | -0.417 | -1.91999 | -0.227 | -1.455 | -0.833 |
| ALPK3 | -0.497 | -0.65822 | 0.094 | -0.676 | 0.63 | -1.078 | -0.103 |
| POLD4 | -0.191 | 0.747825 | 0.817 | 0.981 | 0.526 | -0.198 | 0.661 |
| CTDSP1 | -0.776 | -0.75905 | -0.707 | -1.46302 | -0.168 | -0.736 | -1.14472 |
| OVOL2 | -0.864 | -1.13904 | -0.035 | -1.05788 | -0.67 | -0.452 | -1.7116 |
| VPS16 | 0.484 | 0.339 | 0.377 | 1.09735 | 0.018 | 0.127 | 0.491 |
| LYNX1 | 0.784 | -1.13423 | 0.294 | 0.513 | 0.055 | 0.312 | -0.794 |
| UCK1 | -0.085 | -0.64497 | -0.512 | -0.222 | -0.243 | -0.411 | -0.066 |
| DUOXA1 | -1.757 | -1.34378 | -0.046 | -1.159 | 0.186 | -0.417 | -1.31322 |
| TMEM54 | -1.16 | -1.00933 | 0.123 | -0.669 | -0.239 | -0.693 | -0.977 |
| NOXO1 | -1.408 | -0.895 | -0.466 | -0.341 | 0.035 | -1.812 | -1.44554 |
| TPRA1 | 0.371 | 0.434 | 0.445 | 1.327981 | 0.152 | -0.084 | 1.173489 |
| PPM1M | 0.183 | -0.98931 | -0.375 | -1.19813 | -0.214 | -0.147 | -0.877 |
| GAB3 | 0.393 | | 1.175603 | 0.635 | 0.929359 | 3.165 | 3.047941 |
| BIRC5 | -0.196 | -2.427 | -1.68307 | -0.978 | -1.119 | -1.12051 | -1.058 |
| BARD1 | -0.17 | -2.152 | -1.33631 | -1.275 | -0.316 | -0.057 | -0.831 |
| BRCA1 | -0.491 | -2.45 | -1.34442 | -0.884 | -0.349 | -0.29 | -0.616 |
| BRCA2 | -1.425 | -1.615 | -1.04008 | -0.914 | -0.224 | 0.029 | -0.256 |
| BUB1B | -0.179 | -2.948 | -1.08894 | -1.464 | -0.862 | -0.373 | -1.075 |
| CCNE1 | 0.491 | -1.853 | -1.63262 | -0.655 | -0.424 | 0.217 | -0.105 |
| CDC20 | 0.66496 | -1.891 | -0.431 | -0.623 | -1.04196 | -0.716 | -0.77 |
| CDC25A | -0.358 | -3.403 | -2.87343 | -2.189 | -1.00626 | -0.676 | -1.64 |
| CDC25B | 0.983113 | -0.75 | 0.425 | 0.241 | -0.706 | 0.355 | -0.398 |
| CDKN1A | 2.40859 | 1.827 | 2.51054 | 2.456 | 0.773 | 1.898677 | 2.535 |
| CDKN1B | 0.76127 | -0.71 | -0.116 | 0.127 | -0.002 | 0.008 | -0.131 |
| CDKN2C | 0.650723 | 2.279 | -0.863 | -0.204 | -0.271 | -0.714 | -0.272 |
| CDKN3 | 0.675645 | -1.517 | -0.214 | 0.327 | -0.624 | 0.028 | -0.724 |
| CENPE | -1.51 | -1.741 | -1.086 | -0.753 | -0.745 | -0.233 | -0.68 |
| CHEK1 | -0.245 | -2.39 | -1.59439 | -1.196 | -0.507 | -0.434 | -0.885 |

| | | | | | | | |
|---|---|---|---|---|---|---|---|
| CKS2 | 0.813395 | -1.387 | -0.398 | -0.366 | -0.766 | -0.478 | -0.423 |
| H2AFX | 1.476417 | -0.086 | 0.171 | 0.784 | -0.265 | 0.816 | 0.806 |
| HMMR | -0.275 | -2.409 | -1.34424 | -0.508 | -0.83 | -0.105 | -1.04 |
| HNRNPD | -0.241 | -0.959 | -1.99981 | -1.619 | -0.299 | -0.056 | -1.024 |
| LMNB1 | -0.068 | -3.241 | -1.63934 | -2.16 | -0.754 | -0.671 | -1.541 |
| MAD2L1 | 0.317 | -2.592 | -1.50258 | -1.538 | -0.89 | -0.43 | -0.816 |
| MCM2 | -0.519 | -2.804 | -1.04745 | -0.744 | -0.467 | -0.913 | -0.654 |
| MCM3 | -0.154 | -2.131 | -1.09519 | -1.04 | -0.296 | -0.394 | -0.788 |
| MCM5 | -0.69706 | -2.862 | -1.21159 | -1.178 | -0.316 | -0.797 | -0.977 |
| MKI67 | -0.193 | -2.944 | -1.24829 | -0.732 | -0.913 | -0.037 | -1.194 |
| MYBL2 | 0.054 | -2.25 | -1.41798 | -0.487 | -0.967 | -0.924 | -1.619 |
| MYC | -0.63859 | -0.668 | -3.65354 | -1.351 | -0.424 | -0.895 | -1.334 |
| NBN | -0.81501 | -0.016 | -0.39 | -0.764 | -0.133 | 0.356 | -0.341 |
| NME1 | -1.06345 | -1.308 | -1.96418 | -1.816 | -0.53569 | -1.12823 | -1.993 |
| PLK1 | -0.021 | -2.879 | -1.6178 | -1.189 | -1.21305 | -0.758 | -1.408 |
| PNN | -0.87971 | -0.279 | -0.17 | -0.689 | -0.032 | 0.592 | -0.452 |
| RFC3 | -0.284 | -2.062 | -1.79621 | -1.303 | -0.708 | -0.647 | -1.047 |
| RRM2 | 0.475 | -3.657 | -1.1451 | -0.75 | -0.696 | -0.182 | -0.313 |
| SRSF1 | -0.282 | -0.535 | -1.28233 | -0.843 | -0.286 | -0.186 | -0.289 |
| SNRPB | -0.163 | -0.657 | -0.201 | -0.643 | 0.533 | -1.17707 | -0.544 |
| SSRP1 | -0.493 | -1.251 | -1.85658 | -1.263 | -0.507 | -0.73 | -1.095 |
| AURKA | -0.339 | -2.317 | -1.62391 | -1.177 | 0.949 | -1.0759 | -1.61 |
| SUV39H1 | -0.331 | -2.313 | -1.89664 | -1.771 | -0.862 | -1.43712 | -1.402 |
| TK1 | 0.605 | -1.331 | -0.162 | 0.28 | -0.373 | -0.717 | -0.345 |
| TMPO | -0.232 | -2.082 | -1.19817 | -1.626 | -0.525 | 0.448 | -1.245 |
| TOP2A | -0.41 | -2.583 | -1.1258 | -0.955 | -0.835 | -0.439 | -0.956 |
| TUBG1 | 0.603 | -0.295 | -0.978 | -0.164 | -0.202 | 0.189 | -0.322 |
| UNG | 0.093 | -1.901 | -1.305 | -1.255 | -0.398 | -0.681 | -0.815 |
| USP1 | -0.322 | -1.763 | -1.02561 | -1.162 | -0.32 | -0.045 | -0.794 |
| WEE1 | 0.226 | -2.32 | -1.33323 | -1.029 | -0.453 | -0.18 | -0.42 |
| SLBP | 0.881185 | -0.51 | -0.366 | -0.107 | -0.149 | 0.197 | 0.318 |
| CCNB2 | 0.021 | -2.704 | -1.166 | -0.679 | -0.899 | -0.946 | -1.136 |
| AURKB | 0.65829 | -2.813 | -1.36448 | -1.382 | -1.17824 | -0.914 | -0.885 |
| TRIP13 | -0.387 | -2.404 | -1.75184 | -2.029 | -1.12423 | -1.02322 | -1.427 |
| ESPL1 | -0.242 | -3.077 | -1.61956 | -1.809 | -0.847 | -1.37191 | -1.149 |
| GINS1 | -0.288 | -2.54 | -1.5 | -1.791 | -0.501 | -0.872 | -1.415 |
| NUP153 | -0.382 | -0.538 | -1.053 | -0.933 | -0.348 | 0.272 | -0.782 |
| RAD50 | -1.3582 | 0.172 | -0.34 | -0.442 | 0.01 | 0.29 | -0.196 |
| DDX39A | -0.71729 | -0.997 | -1.12218 | -1.04 | -0.799 | -1.36107 | -1.177 |
| STAG1 | -0.76982 | -0.619 | -1.00408 | -0.321 | -0.399 | -0.24 | -0.801 |
| PAICS | -0.576 | -1.398 | -2.33599 | -1.489 | -0.42 | -0.554 | -1.222 |
| RAD51AP1 | -0.367 | -3.323 | -1.55736 | -1.68 | -0.756 | -1.12014 | -1.4 |
| POLD3 | -0.448 | -1.699 | -1.43919 | -1.302 | -0.531 | -0.551 | -1.066 |
| PLK4 | -0.191 | -2.667 | -1.57887 | -1.627 | -0.951 | -0.565 | -1.052 |

| | | | | | | | |
|---|---|---|---|---|---|---|---|
| CHEK2 | 0.136 | -1.937 | -1.67643 | -0.852 | -0.481 | -0.352 | -0.942 |
| EXOSC8 | -0.324 | -1.308 | -1.54909 | -1.116 | -0.532 | -0.669 | -1.101 |
| NUP205 | -0.404 | -1.16 | -1.17672 | -1.142 | -0.3 | -0.687 | -0.958 |
| CBX5 | 0.680288 | -0.832 | -0.303 | 0.448 | 0.167 | 0.654 | 0.433 |
| ORC6 | 0.611145 | -1.612 | -0.234 | -0.651 | -0.661 | -0.095 | -0.439 |
| POLA2 | 0.261 | -1.811 | -1.26454 | -0.4 | -0.348 | -0.683 | -0.207 |
| UBE2S | 1.035672 | -0.683 | 0.242 | 0.028 | -0.649 | -0.152 | -0.263 |
| RACGAP1 | -0.179 | -2.286 | -1.0987 | -1.127 | -0.801 | -0.996 | -0.971 |
| DONSON | 0.326 | -0.948 | -1.36639 | -0.542 | -0.091 | -0.095 | -0.586 |
| TIPIN | -0.60324 | -1.247 | -1.63547 | -0.688 | -0.578 | -0.434 | -1.1 |
| DEPDC1 | -0.699 | -2.839 | -2.088 | -1.352 | -0.974 | -0.777 | -1.552 |
| ASF1B | 0.747 | -2.311 | -0.587 | -0.167 | -0.767 | -0.529 | -0.064 |
| POLE4 | 0.687 | 0.105 | 0.677 | -0.017 | -0.124 | -0.213 | 0.055 |
| SPC25 | -0.184 | -3.514 | -1.96001 | -1.631 | -1.01082 | -1.08042 | -1.397 |
| GINS3 | -0.337 | -2.165 | -1.068 | -1.723 | -0.583 | -0.325 | -1.609 |
| DCLRE1B | -0.362 | -1.753 | -1.316 | -1.44 | -0.744 | -0.738 | -0.721 |
| DSCC1 | 0.497 | -2.461 | -1 | -1.016 | -0.704 | -0.35 | -0.802 |
| DCTPP1 | -0.72901 | -0.785 | -1.29803 | -1.516 | -0.643 | -1.19851 | -0.964 |
| E2F8 | -1.0386 | -4.255 | -1.98806 | -1.854 | -1.41743 | -0.969 | -1.937 |
| ANP32E | -1.006 | -1.511 | -0.739 | -0.33 | -0.491 | -0.222 | -1.329 |
| DIAPH3 | -0.323 | -2.3 | -1.198 | -0.926 | -0.571 | -0.348 | -0.835 |
| CDCA3 | 0.241 | -2.993 | -1.36594 | -1.479 | -1.02487 | -1.19848 | -1.332 |
| GINS4 | 0.731 | -1.132 | -0.725 | 0.113 | -0.43 | -0.478 | -0.074 |
| PHF5A | -0.189 | -0.837 | -1.074 | -0.984 | -0.274 | -0.832 | -0.852 |
| KIF18B | 0.11 | -2.945 | -0.931 | -1.404 | -1.122 | -0.781 | -0.893 |
| SPC24 | -0.097 | -3.64 | -1.7526 | -1.419 | -1.00483 | -1.22201 | -1.168 |
| TUBB | -0.015 | -1.237 | -1.4191 | -1.436 | -0.6 | -0.73 | -1.233 |
| MMS22L | -0.6283 | -2.222 | -1.71673 | -1.728 | -0.521 | -0.548 | -1.238 |
